# Seladelpar efficacy and safety at 3 months in patients with primary biliary cholangitis: ENHANCE, a phase 3, randomized, placebo-controlled study

**DOI:** 10.1097/HEP.0000000000000395

**Published:** 2023-04-06

**Authors:** Gideon M. Hirschfield, Mitchell L. Shiffman, Aliya Gulamhusein, Kris V. Kowdley, John M. Vierling, Cynthia Levy, Andreas E. Kremer, Ehud Zigmond, Pietro Andreone, Stuart C. Gordon, Christopher L. Bowlus, Eric J. Lawitz, Richard J. Aspinall, Daniel S. Pratt, Karina Raikhelson, Maria S. Gonzalez-Huezo, Michael A. Heneghan, Sook-Hyang Jeong, Alma L. Ladrón de Guevara, Marlyn J. Mayo, George N. Dalekos, Joost P.H. Drenth, Ewa Janczewska, Barbara A. Leggett, Frederik Nevens, Victor Vargas, Eli Zuckerman, Christophe Corpechot, Eduardo Fassio, Holger Hinrichsen, Pietro Invernizzi, Palak J. Trivedi, Lisa Forman, David E.J. Jones, Stephen D. Ryder, Mark G. Swain, Alexandra Steinberg, Pol F. Boudes, Yun-Jung Choi, Charles A. McWherter

**Affiliations:** 1 University Health Network and Division of Gastroenterology and Hepatology, Toronto Centre for Liver Disease, University of Toronto, Toronto, Ontario, Canada; 2 Liver Institute of Virginia, Bon Secours Mercy Health, Bon Secours Liver Institute of Richmond, Richmond, Virginia, USA; 3 Bon Secours Liver Institute of Hampton Roads, Newport News, Virginia, USA; 4 University Health Network and Department of Medicine, Toronto Centre for Liver Disease, University of Toronto, Toronto, Ontario, Canada; 5 Liver Institute Northwest, Seattle, Washington, USA; 6 Departments of Medicine and Surgery, Baylor College of Medicine, Houston, Texas, USA; 7 Schiff Center for Liver Diseases, University of Miami Miller School of Medicine, Miami, Florida, USA; 8 Department of Gastroenterology and Hepatology, University Hospital Zürich, Zürich, Switzerland; 9 Center for Autoimmune Liver Diseases, Tel-Aviv Sourasky Medical Center and Sackler Faculty of Medicine, Tel-Aviv University, Tel-Aviv, Israel; 10 Department of Medical and Surgical Sciences, Division of Internal Medicine, Maternal-Infantile and Adult, University of Modena and Reggio Emilia, Modena, Italy; 11 Postgraduate School of Allergy and Clinical Immunology, University of Modena and Reggio Emilia, Italy; 12 Division of Hepatology, Henry Ford Hospital, Wayne State University School of Medicine, Detroit, Michigan, USA; 13 Division of Gastroenterology and Hepatology, University of California Davis School of Medicine, Sacramento, California, USA; 14 Texas Liver Institute, University of Texas Health San Antonio, San Antonio, Texas, USA; 15 Department of Hepatology, Portsmouth Liver Centre, Portsmouth Hospitals National Health Service Trust, Queen Alexandra Hospital, Portsmouth, UK; 16 Autoimmune and Cholestatic Liver Center, Massachusetts General Hospital, Boston, Massachusetts, USA; 17 Saint Petersburg State University, St. Petersburg, Russia; 18 City Hospital 31, St. Petersburg, Russia; 19 Metepec Edo Mex., Mexico; 20 King’s College Hospital National Health Service Foundation Trust, London, UK; 21 Department of Internal Medicine, Seoul National University Bundang Hospital, Seongnam, Korea; 22 Center of Research and Gastroenterology, Mexico City, Mexico; 23 Division of Digestive and Liver Diseases, University of Texas Southwestern, Dallas, Texas, USA; 24 Department of Medicine and Research Laboratory of Internal Medicine, National Expertise Center of Greece in Autoimmune Liver Diseases, European Reference Network on Hepatological Diseases (ERN RARE-LIVER), General University Hospital of Larissa, Larissa, Greece; 25 Department of Gastroenterology and Hepatology, Radboudumc, Nijmegen, The Netherlands; 26 Department of Basic Medical Sciences, Faculty of Health Sciences in Bytom, Medical University of Silesia, Katowice, Poland; 27 ID Clinic, Myslowice, Poland; 28 School of Medicine, University of Queensland, Herston, Queensland, Australia; 29 University Hospitals KU Leuven, Belgium; 30 Center of European Reference Network (ERN) RARE-LIVER, Leuven, Belgium; 31 Liver Unit, Hospital Universitari Vall d’Hebron, Universitat Autònoma de Barcelona, Bellaterra, Spain; 32 Centro de Investigación Biomédica en Red de Enfermedades Hepáticas y Digestivas (CIBEREHD), Barcelona, Spain; 33 Liver Unit, Carmel Medical Center, Technion, Faculty of Medicine, Israeli Association for the Study of the Liver, Haifa, Israel; 34 Reference Center for Inflammatory Biliary Diseases and Autoimmune Hepatitis, Hepatology and Gastroenterology Department (MIVB-H), Filière Maladies Rares: Maladies Rares du Foie de l’Adulte et de l’Enfant (FILFOIE), European Reference Network (ERN) RARE-LIVER, Inserm, Centre de Recherche Saint-Antoine (CRSA), Assistance Publique-Hopitaux of Paris (AP-HP), Saint-Antoine Hospital, Sorbonne Universités, Paris, France; 35 DIM Clínica Privada, Ramos Mejía, Buenos Aires province, Argentina; 36 Gastroenterology–Hepatology Center Kiel, Kiel, Germany; 37 Department of Medicine and Surgery, Center for Autoimmune Liver Diseases, University of Milano-Bicocca, Monza, Italy; 38 Division of Gastroenterology, Fondazione IRCCS San Gerardo dei Tintori & European Reference Network on Hepatological Diseases (ERN RARE-LIVER), Monza, Italy; 39 National Institute for Health Research (NIHR), Birmingham Biomedical Research Centre, Centre for Liver and Gastroenterology Research, University of Birmingham, UK; 40 Liver Unit, University Hospitals Birmingham Queen Elizabeth, Birmingham, UK; 41 Institute of Immunology and Immunotherapy, University of Birmingham, UK; 42 Institute of Applied Health Research, University of Birmingham, UK; 43 University of Colorado, Aurora, Colorado, USA; 44 Institute of Cellular Medicine and National Institute for Health Research (NIHR) Newcastle Biomedical Research Centre, Newcastle University, Newcastle upon Tyne, UK; 45 National Institute for Health Research (NIHR) Nottingham Biomedical Research Centre at Nottingham University Hospitals National Health Service (NHS) Trust and the University of Nottingham, Queens Medical Centre, Nottingham, UK; 46 Department of Medicine, University of Calgary, Calgary, Alberta, Canada; 47 CymaBay Therapeutics, Newark, California, USA

## Abstract

ENHANCE was a phase 3 study that evaluated efficacy and safety of seladelpar, a selective peroxisome proliferator-activated receptor-δ (PPAR) agonist, versus placebo in patients with primary biliary cholangitis with inadequate response or intolerance to ursodeoxycholic acid (UDCA).

**Approach and Results:**

Patients were randomized 1:1:1 to oral seladelpar 5 mg (n=89), 10 mg (n=89), placebo (n=87) daily (with UDCA, as appropriate). Primary end point was a composite biochemical response [alkaline phosphatase (ALP) < 1.67×upper limit of normal (ULN), ≥15% ALP decrease from baseline, and total bilirubin ≤ ULN] at month 12. Key secondary end points were ALP normalization at month 12 and change in pruritus numerical rating scale (NRS) at month 6 in patients with baseline score ≥4. Aminotransferases were assessed. ENHANCE was terminated early following an erroneous safety signal in a concurrent, NASH trial. While blinded, primary and secondary efficacy end points were amended to month 3. Significantly more patients receiving seladelpar met the primary end point (seladelpar 5 mg: 57.1%, 10 mg: 78.2%) versus placebo (12.5%) (*p* < 0.0001). ALP normalization occurred in 5.4% (*p*=0.08) and 27.3% (*p* < 0.0001) of patients receiving 5 and 10 mg seladelpar, respectively, versus 0% receiving placebo. Seladelpar 10 mg significantly reduced mean pruritus NRS versus placebo [10 mg: −3.14 (*p*=0.02); placebo: −1.55]. Alanine aminotransferase decreased significantly with seladelpar versus placebo [5 mg: 23.4% (*p*=0.0008); 10 mg: 16.7% (*p*=0.03); placebo: 4%]. There were no serious treatment-related adverse events.

**Conclusions:**

Patients with primary biliary cholangitis (PBC) with inadequate response or intolerance to UDCA who were treated with seladelpar 10 mg had significant improvements in liver biochemistry and pruritus. Seladelpar appeared safe and well tolerated.

## Graphical abstract

**Figure:**
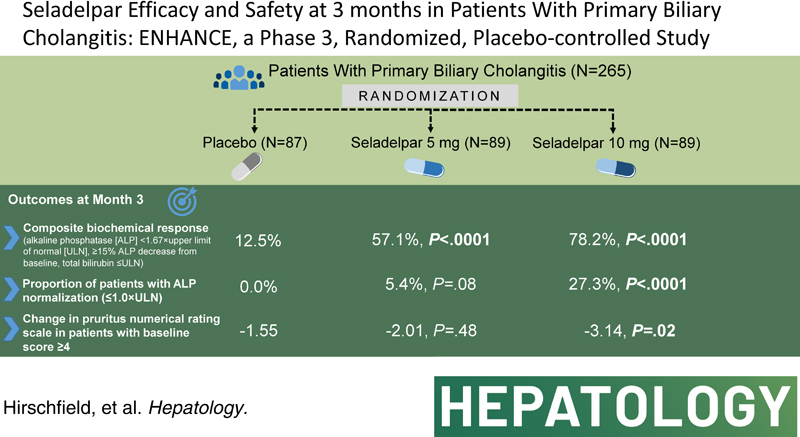

## INTRODUCTION

People living with primary biliary cholangitis (PBC) frequently have impaired quality and quantity of life, resulting from a chronic autoimmune-mediated, cholestatic, and fibrosing liver injury.^1,2^ PBC is a rare, progressive in nature, and usually female-predominant liver disease.^1,2^ Cholestatic liver injury is characterized by variable rates of immune-mediated destruction of intrahepatic bile ducts accompanied by portal inflammation.^3–6^ Bile duct loss (ductopenia) leads to cholestasis and hepatocellular injury and a progressive liver injury with fibrosis, end-stage liver disease, and, ultimately, liver failure.^4,6–8^ Independent of disease stage, many people living with PBC experience significant impairment to quality of life, particularly from fatigue and pruritus.^2,3,7^

Histologic damage characterized by a granulomatous lymphocytic cholangitis is associated with abnormal serum liver tests, including elevated serum alkaline phosphatase (ALP), gamma-glutamyl transferase (GGT), aminotransferase activity, and total bilirubin.^9^ These biochemical indices of disease correlate with disease severity, treatment efficacy, and outcomes.^10–14^ Elevated ALP and total bilirubin [1.67× upper limit of normal (ULN) and 1×ULN, respectively] have been incorporated into clinical end points because they serve as surrogates of disease activity related to risk for disease progression that are reasonably likely to predict clinical benefit.^11,15^ Recent analyses demonstrating that outcomes for patients are improved with normal ALP and total bilirubin emphasize the importance of normalizing serum liver tests.^13^ Treatment of PBC focuses on initial use of ursodeoxycholic acid (UDCA) followed by add-on therapy with the conditionally approved farnesoid X receptor (FXR) agonist obeticholic acid or off-label fibrates, such as bezafibrate, in patients not having a satisfactory biochemical response.^5,6,16^ There is no approved therapy for fatigue, and pruritus is managed with limited success. Obeticholic acid and other FXR agonists have been noted to exacerbate pruritus in some patients.^15^

Seladelpar is a potent and selective peroxisome proliferator-activated receptor (PPARδ) agonist that has been shown to decrease levels of biochemical markers of cholestasis, liver injury, and inflammation in patients with PBC.^17–19^ In a previous phase 2 study in patients with PBC, at 12 weeks, seladelpar [50 or 200 mg once daily (QD)] reduced ALP >50%; however, the study was stopped early due to rapid onset of asymptomatic, reversible elevations in hepatic aminotransferases in 3 patients.^18^ A subsequent phase 2, open-label, dose-ranging study evaluated efficacy and safety of seladelpar at doses of 2, 5, or 10 mg QD in patients with PBC, with increases in dose allowed up to 10 mg QD based on biochemical response.^19^ Seladelpar was safe and well tolerated in this study, and the composite biochemical end point was achieved at 1 year in 64%, 53%, and 67% of patients randomized to 2, 5, and 10 mg/d, respectively. Improvement in pruritus was also observed.^20^

The objective of this phase 3 study was to evaluate the safety of seladelpar 5 and 10 mg QD and to assess its effect on ALP, total bilirubin, biochemical markers of disease, and pruritus in patients with PBC at high risk for disease progression. The study originally aimed to evaluate treatment through 12 months; however, it was terminated early due to unexpected histological findings (ie, portal inflammation and interface hepatitis with plasma cells, bile duct injury/cholangitis, vascular changes, and other miscellaneous findings) in a concurrent study of seladelpar in patients with NASH (NCT03551522). A detailed independent investigation by a committee of pathologists and hepatologists determined that the histological findings following seladelpar treatment in the NASH trial did not differ qualitatively from baseline and were unrelated to seladelpar.^21^ The existence of these findings in biopsies of patients with NASH was confirmed in a subsequent literature report.^22^ The time points for end point analysis were, therefore, adjusted to 3 months before unblinding. The results of these analyses for ENHANCE are presented here.

## METHODS

### Patients

Patients aged 18 to 75 years diagnosed with PBC [≥2 of the following criteria: history of ALP > ULN for ≥6 months, positive antimitochondrial antibody titers (>1/40 on immunofluorescence or M2-positive by ELISA) or PBC-specific antinuclear antibodies, or documented liver biopsy histology consistent with PBC] were screened for eligibility. Patients with ALP ≥1.67×ULN and total bilirubin ≤2×ULN were eligible.^15^ Patients must have been receiving a stable and recommended UDCA dose (generally 13–15 mg/kg/d^6^) for the prior 12 months unless they were UDCA intolerant. UDCA treatment continued in patients receiving UDCA before study enrollment. Exclusion criteria included aspartate aminotransferase (AST) or alanine aminotransferase (ALT) >3×ULN, advanced PBC as defined by the Rotterdam criteria (coincident albumin less than the lower limit of normal and total bilirubin >1×ULN), creatine kinase >1×ULN, estimated glomerular filtration rate <60 mL/min/1.73 m^2^, international normalized ratio >1×ULN, circulating platelet count of <100×10^3^/µL, clinically significant hepatic decompensation or presence of another chronic liver disease, or any other medical condition that would compromise safety or confound study results. Patients with cirrhosis who did not have a history or current evidence of hepatic decompensation but met all other criteria were eligible. Use of obeticholic acid, fibrates, or experimental or unapproved therapies <30 days before screening; colchicine, methotrexate, azathioprine, or systemic corticosteroids for >2 weeks within 2 months before screening; or simvastatin <7 days before screening was prohibited.

### Study design

This phase 3, double-blind, randomized, placebo-controlled study was conducted at 111 sites in 21 countries. The protocol was approved by appropriate local and national institutional review boards or independent ethics committees, and the trial was conducted in accordance with the Declaration of Helsinki and Good Clinical Practice guidelines. All patients provided written informed consent. The trial was preregistered (www.clinicaltrials.gov; NCT03602560). Pol Boudes, Christopher L. Bowlus, Gideon M. Hirschfield, Cynthia Levy, Marlyn J. Mayo, Alexandra Steinberg, John M. Vierling, Charles A. McWherter, and Yun-Jung Choi provided input to study design that led to the protocol. Gideon M. Hirschfield, Charles McWherter, and Yun-Jung Choi had access to all data and reviewed and can vouch for the integrity of the data analyses.

#### Original study design

The study was initially designed as a 12-month study where eligible patients were centrally randomized 1:1:1 through an interactive voice/web response system to receive seladelpar (CymaBay Therapeutics, Inc., Newark, CA) 5 or 10 mg QD orally or matching placebo following a 2-week screening period and subsequent 2-week run-in period. Patients and investigators were blinded to treatment, and blinding was maintained using a matched placebo. Patients were stratified by ALP level (<350 or ≥350 U/L) and pruritus numerical rating scale (NRS) (< 4 or ≥ 4). Patients receiving seladelpar 5 mg were to be uptitrated to 10 mg QD if they had not met the primary end point at month 6. Randomization occurred between November 26, 2018, and November 12, 2019.

#### Amended analysis plan

Because of unexpected histological findings in a concurrent phase 2 study of seladelpar in patients with NASH, dosing in ENHANCE was interrupted on November 25, 2019, and the study was terminated prematurely on December 20, 2019. The biopsy tissues and full clinical profile of each patient in the NASH trial were reviewed by an independent committee of pathologists and hepatologists who concluded that the histological features of concern were also observed in baseline biopsies and were unrelated to seladelpar treatment.^21^ At the time of termination, ENHANCE was fully enrolled, and patients had a broad range of study drug treatment durations. Patients were requested to discontinue treatment and return to their study site for a safety follow-up visit. While still blinded, all end points were amended to month 3 [previous primary and key secondary end point times were month 12 (for biochemical responses) or month 6 (for pruritus)].

### Study assessments

Study assessment visits were performed at screening; run-in; day 1 (randomization); months 1, 3, and 6; and at a follow-up visit 4 weeks after the end of treatment. Fasting blood samples were obtained at each visit for ALP, total bilirubin, other biochemistry, lipids, bile acid precursor 7α-hydroxy-4-cholesten-3-one (C4), IgM, and hematology assays. Patients recorded a daily pruritus NRS each evening for the previous 24 hours on a scale of 0 (no itching) to 10 (worst imaginable itching) using an e-diary from the run-in visit through the month 6 visit.

Safety assessments included physical examinations, vital sign evaluations, and laboratory tests. Treatment-emergent adverse events (TEAEs) were summarized using Medical Dictionary for Regulatory Activities version 21.0 and graded using National Cancer Institute Common Terminology Criteria for Adverse Events version 5.0.

### Primary end point

The primary end point was a composite biochemical response defined as ALP <1.67×ULN, ≥15% ALP decrease from baseline, and total bilirubin ≤ ULN^15^ at month 3.

### Secondary end points

Key secondary end points were the proportion of patients with ALP ≤1.0×ULN (normalization) at month 3 and the change in pruritus NRS from baseline at month 3 in patients with baseline pruritus NRS ≥4, which was considered symptomatic.^23^ The composite biochemical end point, ALP normalization, and change from baseline in pruritus NRS were also evaluated at month 6 as secondary end points. Other secondary end points included ALP <1.5×ULN; other published PBC treatment response criteria;^9,24–27^ mean absolute and least squares (LS) mean relative (percent) change from baseline in ALP, ALT, AST, total bilirubin, GGT, and lipid levels; the 5-domain itch scale; and the PBC-40 quality-of-life questionnaire. Exploratory end points were LS mean relative (percent) changes from baseline in C4 and IgM serum levels at month 3. Post hoc analyses included the proportions of patients with baseline ALT elevations who normalized at month 3, with normal baseline ALT levels who had elevations at month 3, and with baseline pruritus NRS ≥4 who had pruritus NRS decreases of at least 2, 3, or 4 points at month 3. NRS categories were as follows: 0=no pruritus, 1–3=mild pruritus, 4–6=moderate pruritus, 7–8=severe pruritus, and ≥9=very severe pruritus.^23^

### Statistical analysis

A sample size of 80 patients per treatment group, or 240 patients total, was estimated to provide (1) >90% power to detect a difference in the composite end point response rate (based on estimated response rates of <15%^15^ for placebo and 40% for seladelpar 10 mg), (2) 30% difference in normalization of ALP response rate (based on an estimated response rate of 5% for placebo), and (3) ≥3-point difference in pruritus NRS score between the seladelpar 10 mg and placebo groups. When the study was terminated and while still blinded, it was determined that the month 3 time point had a sufficient number of patients with available data to provide adequate power to detect differences in the primary and key secondary end points. A sample size of at least 53 patients per treatment group provided >83% power to detect a 25% difference in the composite end point response rate between the seladelpar 10 mg and placebo groups, >90% power to detect a 30% difference in the ALP normalization response rate between the seladelpar 10 mg and placebo groups, and ≥88% power to detect a ≥3-point difference in pruritus NRS score between the seladelpar 10 mg and placebo groups.

Efficacy was assessed in the modified intent-to-treat (mITT) population, which included all randomized patients who received ≥ 1 dose of study drug, and was analyzed based on randomized treatment. Efficacy analyses were conducted on the (mITT) population excluding patients who discontinued treatment before the month 3 assessment due to study closure. Patients who discontinued treatment before an assessment time point due to reasons other than study closure and did not have an assessment at the specified time point were classified as nonresponders. Safety was assessed during the study in all randomized patients who received ≥1 dose of study drug and was analyzed based on treatment received.

Statistical comparisons of efficacy end points included the seladelpar 10- and 5-mg groups versus the placebo group and the seladelpar 10-mg group versus the 5-mg group for the primary and key secondary end points and mean change from baseline in ALP. Statistical tests were conducted using 2-sided tests at a 0.05 level of significance. The primary end point and ALP normalization response rate were analyzed using the Cochran-Mantel-Haenszel test adjusted for randomization stratification variables. Baseline pruritus NRS was defined as the mean of daily recorded scores during the run-in period. Assessment of pruritus NRS change from baseline compared the weekly averaged pruritus NRS of patients with a baseline score ≥4 using an analysis of covariance (ANCOVA) model. Least squares mean percent change from baseline for serum biochemistries was estimated using an ANCOVA model with percent change from baseline as the dependent variable, treatment group and randomization stratification factors as fixed effects, and baseline as a covariate.

## RESULTS

### Patient disposition

**FIGURE 1:**
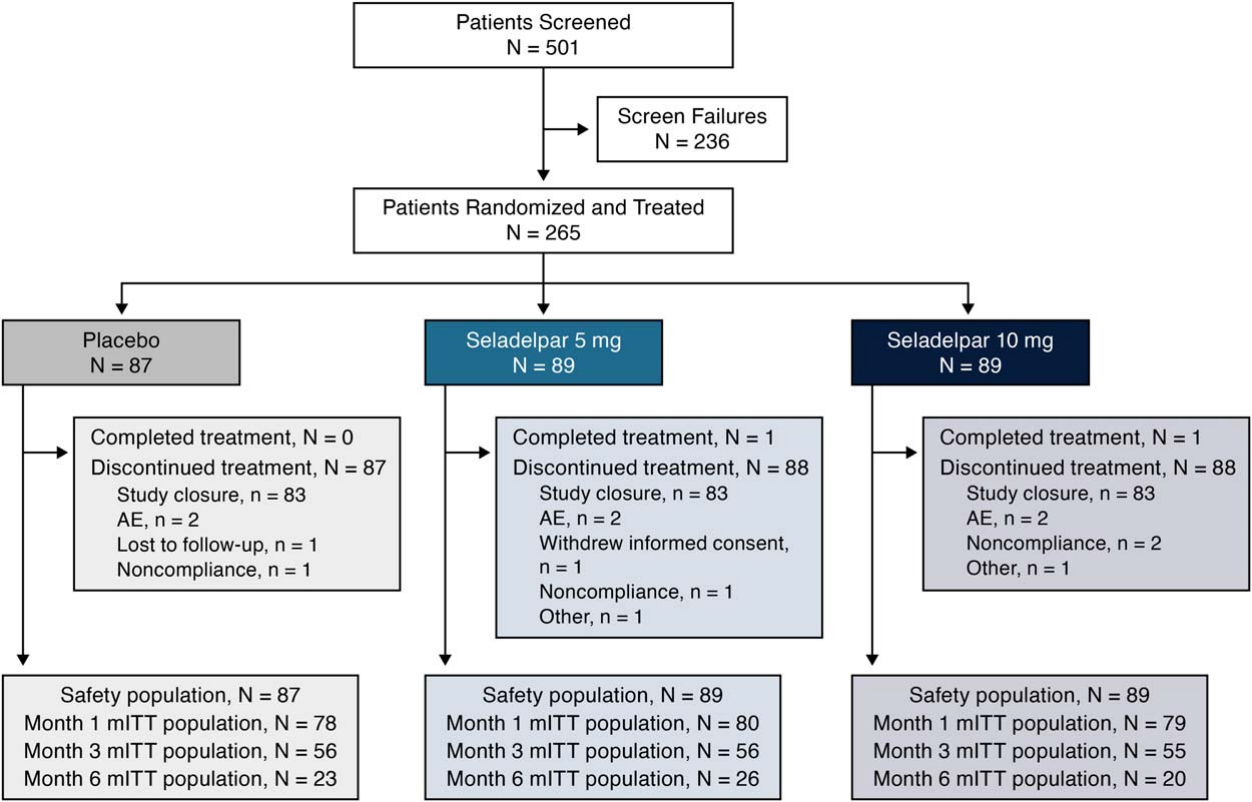
Patient flowchart. Screen failures (a patient may be counted in >1 reason for failure): alkaline phosphatase < 1.67× upper limit of normal (ULN) n=149, estimated glomerular filtration rate <60 mL/min/1.73 m^2^ n=27, alanine aminotransferase >3×ULN n=11, total bilirubin > 2.0×ULN n=11, aspartate aminotransferase >3×ULN n=10, did not meet primary biliary cholangitis (PBC) diagnosis criteria n=8, not on a stable and recommended dose of ursodeoxycholic acid (UDCA) for past 12 months OR intolerant to UDCA (last dose >3 months before screening) n=7, creatine kinase >1.0×ULN n=6, platelet count <100×10^3^/µL n=6, had advanced PBC per Rotterdam criteria n=4, international normalized ratio >1.0×ULN n=4, presence of clinically significant hepatic decompensation n=4, presence of chronic liver disease n=4, presence of any other condition that would compromise patient safety/clinical trial quality n=4, did not provide written informed consent n=4, evidence of drug abuse n=3, use of fibrates within 30 days before screening n=2, use of simvastatin within 7 days before screening n=2. Abbreviations: AE, adverse event; mITT, modified intent-to-treat; N, number of patients assigned to the treatment group; n, number of patients in the category.

Among 501 patients screened, 265 were randomized to receive placebo (n=87) or seladelpar 5 mg (n=89) or 10 mg (n=89). Two patients completed study treatment through month 12; 255 of 265 (96.2%) patients discontinued treatment due to study closure, 6 (2.3%) discontinued due to TEAEs, 1 (0.4%) withdrew consent, and 1 (0.4%) was lost to follow-up (Figure 1). A total of 237 patients were analyzed for the month 1 treatment time point (placebo: 78; seladelpar 5 mg: 80; and seladelpar 10 mg: 79), 167 were analyzed for the month 3 treatment time point (placebo: 56; seladelpar 5 mg: 56; and seladelpar 10 mg: 55), and 69 were analyzed for the month 6 treatment time point (placebo: 23; seladelpar 5 mg: 26; and seladelpar 10 mg: 20).

Baseline demographics and disease characteristics, including biochemical markers of cholestasis, were well balanced among treatment groups (Table 1). Mean ALP and total bilirubin levels were 291.5 U/L (2.5×ULN) and 0.73 mg/dL (0.66×ULN), respectively; 24% of patients had ALP levels ≥ 350 U/L (3×ULN), and 12% had total bilirubin levels > 1×ULN. Mean ALT level was 46.4 U/L (1.1×ULN). In addition, 31% of patients had a baseline pruritus NRS score ≥4, with a mean score of 6.1; 11% had cirrhosis according to the investigator’s judgment (based on biopsy, liver stiffness, or imaging); 89% were positive for antimitochondrial antibodies; and 6% were UDCA intolerant. Mean total daily UDCA dose was 15.3 mg/kg in patients taking UDCA. Overall, 15% and 9% of patients had previously used obeticholic acid or fibrates, respectively.

**TABLE 1:** Patient baseline demographics and clinical characteristics

	Placebo (N=87)	Seladelpar 5 mg (N=89)	Seladelpar 10 mg (N=89)	Total (N=265)
Age (y)	55.9 (8.2)	54.7 (9.7)	55.6 (9.1)	55.4 (9.0)
Female, n (%)	85 (98)	82 (92)	83 (93)	250 (94)
Race, n (%)				
White	80 (92)	83 (93)	77 (87)	240 (91)
Other^a^	7 (8)	6 (7)	12 (13)	25 (9)
Body mass index (kg/m^2^)	28.2 (5.5)	27.7 (6.1)	27.6 (5.9)	27.8 (5.8)
Duration of PBC (y)	8.4 (6.2)	8.3 (6.4)	8.4 (6.4)	8.4 (6.3)
UDCA				
Use at baseline, n (%)	85 (98)	83 (93)	81 (91)	249 (94)
Total daily dose (mg/kg)	15.0 (2.6)	15.6 (4.4)	15.3 (3.7)	15.3 (3.6)
Minimum, maximum (mg/kg)	10.0, 23.4	7.3, 36.1	7.5, 26.7	7.3, 36.1
ALP (U/L)	293.4 (106.2)	290.5 (104.2)	290.8 (109.1)	291.5 (106.1)
≥350 U/L (3×ULN), n (%)	19 (22)	22 (25)	23 (26)	64 (24)
Total bilirubin (mg/dL)	0.71 (0.32)	0.76 (0.35)	0.72 (0.32)	0.73 (0.33)
>1×ULN, n (%)	9 (10)	13 (15)	9 (10)	31 (12)
ALT (U/L)	44.4 (20.7)	47.7 (21.0)	46.9 (20.8)	46.4 (20.8)
AST (U/L)	37.5 (16.8)	40.1 (14.5)	40.3 (14.9)	39.3 (15.4)
GGT (U/L)	228.9 (193.0)	231.3 (212.0)	243.1 (227.7)	234.5 (210.8)
Pruritus history, n (%)	57 (66)	66 (74)	65 (73)	188 (71)
Pruritus NRS	2.9 (2.5)	2.8 (2.5)	2.7 (2.6)	2.8 (2.6)
≥4, n (%)	27 (31)	27 (30)	27 (30)	81 (31)
≥4	6.1 (1.2)	6.1 (1.4)	6.2 (1.4)	6.1 (1.3)
Antimitochondrial antibodies, n (%)				
Positive	75 (86)	79 (89)	81 (91)	235 (89)
Negative	9 (10)	8 (9)	8 (9)	25 (9)
Equivocal	3 (3)	2 (2)	0	5 (2)
Cirrhosis, n (%)	7 (8)	9 (10)	13 (15)	29 (11)
Prior PBC medications^b^				
UDCA	87 (100)	89 (100)	89 (100)	265 (100)
Obeticholic acid	11 (13)	13 (15)	16 (18)	40 (15)
Fibrates	8 (9)	9 (10)	6 (7)	23 (9)
Other^c^	17 (20)	8 (9)	10 (11)	35 (13)

*Note:* modified intent-to-treat (mITT) population. All values are mean (SD) unless specified otherwise.

a Includes American Indian or Alaska Native, Asian, and Black or African American.

b All listed medications except UDCA were discontinued before study entry.

c Steroids, immunosuppressants, methotrexate, systemic steroids, and colchicine.

Abbreviations: ALP, alkaline phosphatase; ALT, alanine aminotransferase; AST, aspartate aminotransferase; GGT, gamma-glutamyl transferase; n, number of patients in the category; N, number of patients in the treatment group; NRS, numerical rating scale; PBC, primary biliary cholangitis; UDCA, ursodeoxycholic acid; ULN, upper limit of normal.

### Efficacy

#### Composite biochemical response and ALP normalization

**FIGURE 2:**
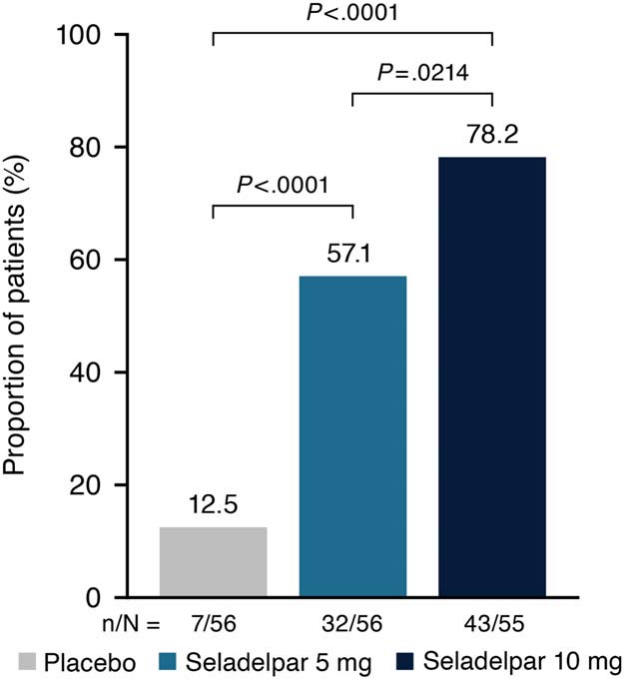
Proportion of patients who achieved the composite biochemical end point at month 3. Composite end point was defined as alkaline phosphatase (ALP) serum levels < 1.67× upper limit of normal (ULN), ≥15% decrease in ALP serum levels, and total bilirubin serum levels ≤ ULN. *p*-values are based on the Cochran-Mantel-Haenszel test adjusted for both randomization stratification variables. Patients who discontinued treatment before month 3 due to reasons other than study termination and who did not have evaluable data at month 3 were considered nonresponders.

At month 3, significantly greater proportions of patients in the seladelpar 5-mg (57.1%, *p* < 0.0001) and 10-mg (78.2%, *p* < 0.0001) groups achieved the primary composite biochemical end point compared with placebo (12.5%) (Figure 2). The composite response rate was significantly greater in the seladelpar 10-mg group versus the 5-mg group (*p*=0.02). Similarly, compared with placebo, the proportion of patients who achieved the composite end point was significantly greater in the seladelpar 5-mg and 10-mg groups at months 1 (*p* < 0.0001 for both) and 6 (*p*=0006 and *p*=0.002, respectively) and significantly greater in the seladelpar 10-mg group versus 5-mg group at month 1 (*p*=0.02) (Supplemental Figure S1, http://links.lww.com/HEP/F475). Among patients with cirrhosis at baseline with available data at month 3, the composite response rate was greater in the seladelpar 5-mg [33.3% (2/6); *p*=0.27] and 10-mg [83.3% (5/6), *p*=0.03] groups versus the placebo group [0% (0/5)]. Although the number of patients was too small to make reliable group comparisons, among UDCA-intolerant patients, the composite response rate at month 3 was numerically greater in the seladelpar 10-mg group (75.0%, n=8) than in the placebo (0.0%, n=2) and seladelpar 5-mg groups (0.0%, n=6) (*p*-values not calculated).

**FIGURE 3:**
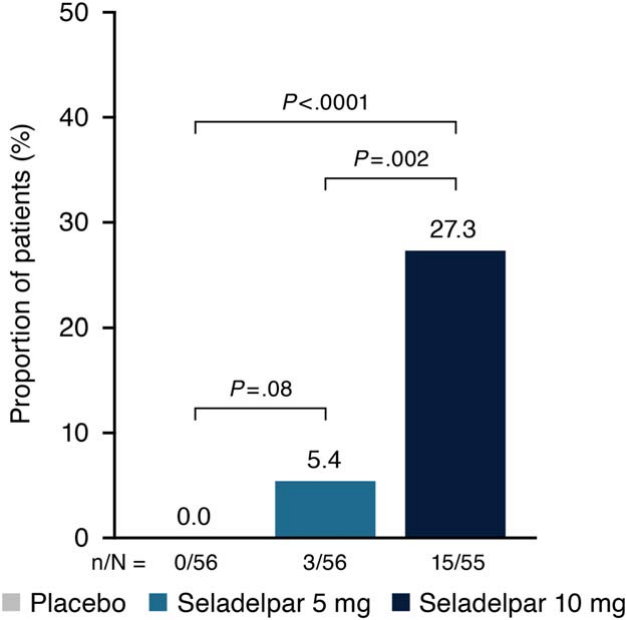
Proportion of patients who achieved alkaline phosphatase (ALP) normalization at month 3 ALP normalization. ALP normalization was defined as serum levels ≤1.0×upper limit of normal. *p*-values are based on the Cochran-Mantel-Haenszel test adjusted for both randomization stratification variables. Patients who discontinued treatment before month 3 due to reasons other than study termination and who did not have evaluable data at month 3 were considered nonresponders.

The proportion of patients attaining ALP normalization at month 3 (≤1.0×ULN) was greater in the seladelpar 10-mg (27.3%) group versus the placebo (0%, *p* < 0.0001) and 5-mg (5.4%, *p*=0.002) groups (Figure 3). In addition, response rates of patients assessed using published PBC response criteria were greater in the seladelpar 5-mg and 10-mg groups at month 3 compared with the placebo group (Supplemental Table S1, http://links.lww.com/HEP/F475).

Examining the individual composite end point components, at month 3, 18% of patients in the placebo group, 64% in the seladelpar 5-mg group, and 82% in the seladelpar 10-mg group achieved ALP < 1.67×ULN, and 23%, 95%, and 95%, respectively, achieved a ≥ 15% decrease from baseline in ALP (Supplemental Table S2, http://links.lww.com/HEP/F475). The response rates for the total bilirubin component (≤ULN) at month 3 were similar among all treatment groups (placebo: 91%; seladelpar 5 mg: 86%; and seladelpar 10 mg: 93%). The proportion of patients with elevated total bilirubin (>ULN) decreased from baseline to month 3 in both seladelpar groups (from 13.0% to 11.1% in the seladelpar 5-mg group and from 5.7% to 3.8% in the seladelpar 10-mg group) but increased from 7.1% to 8.9% in the placebo group. The proportions of patients with ALP <1.5×ULN in the placebo and seladelpar 5-mg and 10-mg groups were 9%, 59%, and 75%, respectively.

#### Pruritus

**FIGURE 4:**
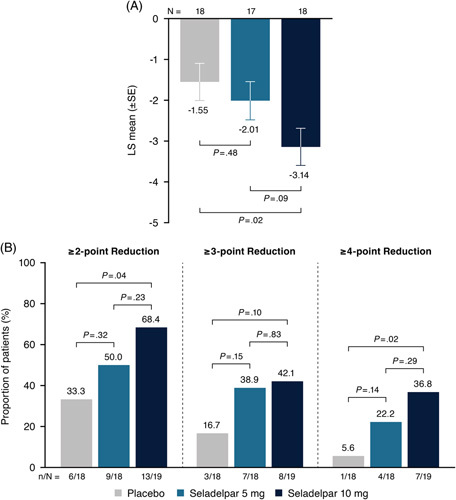
Absolute LS mean (SE) change from baseline in pruritus numerical rating scale (NRS) at month 3 (A) and proportion of patients who achieved point reductions from baseline in pruritus NRS at month 3 (B). For (A) and (B), populations included only patients with pruritus NRS ≥4 at baseline. For (A), change from baseline was estimated by an analysis of covariance model with treatment group and randomization alkaline phosphatase stratification as factors and baseline pruritus score as a covariate. For (B), patients who discontinued treatment before month 3 due to reasons other than study termination and who did not have evaluable data at month 3 were considered nonresponders. *p*-values are per Cochran-Mantel-Haenszel test adjusted for both randomization stratification variables. Abbreviations: LS, least squares; N, number of patients in the treatment group; n, number of patients in the category.

Among patients in the prespecified subgroup with moderate-to-severe pruritus (pruritus NRS ≥4) at baseline who were evaluable at month 3 (placebo, n=18; seladelpar 5 mg, n=17; and seladelpar 10 mg, n=18), the mean decrease from baseline in pruritus NRS was significantly greater in the seladelpar 10-mg (−3.14, *p*=0.02) group versus placebo (−1.55) but not in the 5-mg group (−2.01, *p*=0.48) (Figure 4A). Few patients in this subgroup were evaluable at month 6 (placebo, n=6; seladelpar 5 mg, n=9; and seladelpar 10 mg, n=7), but patients in the 10-mg group had a decrease in pruritus NRS that was greater than that in the placebo and seladelpar 5-mg groups. No significant differences in NRS between the seladelpar 5-mg and placebo groups were noted at any time point (Supplemental Figure S2, http://links.lww.com/HEP/F475). At month 3, dose-ordered increases in the proportion of patients achieving a ≥2-point, ≥3-point, or ≥4-point reduction in pruritus NRS were observed (Figure 4B). A ≥4-point reduction in pruritus NRS was achieved by >1 in 3 (36.8%) patients in the seladelpar 10-mg group compared with ~1 in 20 (5.6%) patients in the placebo group. The differences between the placebo and seladelpar 10-mg groups were significant for the ≥2-point (*p*=0.04) and ≥4-point (*p*=0.02) reductions. A post hoc analysis of these data demonstrated that 27.8% of patients in the placebo group with pruritus NRS ≥4 at baseline had a pruritus NRS score <4 at month 3 compared with 47.1% and 61.1% in the seladelpar 5- and 10-mg groups, respectively (Supplemental Table S3, http://links.lww.com/HEP/F475). Conversely, 2.5% of patients in the placebo group with pruritus NRS <4 at baseline had a pruritus NRS score ≥4 at month 3 compared with 2.4% and 7.3% in the seladelpar 5- and 10-mg groups, respectively.

Greater changes from baseline with seladelpar versus placebo at months 1 and 3 in the 5-domain itch scale total and individual domain scores (Supplemental Figure S3A–L, http://links.lww.com/HEP/F475) and the PBC-40 itch domain score (Supplemental Figure S4A, B, http://links.lww.com/HEP/F475) among patients with baseline NRS ≥ 4 were also observed. The PBC-40 total and individual domain scores did not change with treatment over 3 months (Supplemental Figure S4C–N, http://links.lww.com/HEP/F475).

#### Liver biochemistry and lipids

**FIGURE 5:**
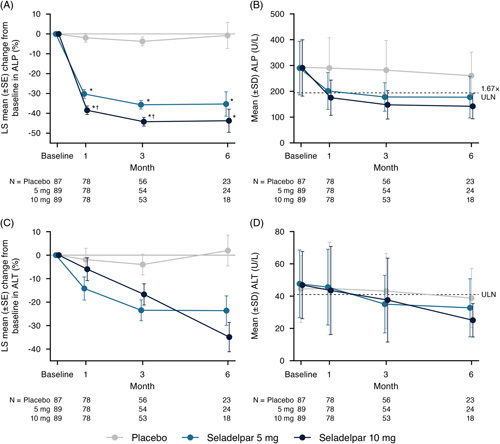
LS mean relative (percent) change from baseline and mean absolute values for ALP (A and B) and ALT (C and D) through month 6. *p < 0.0001 versus placebo, †*p* ≤ 0.002 versus seladelpar 5-mg group. Abbreviations: ALP, alkaline phosphatase; ALT, alanine aminotransferase; LS, least squares; ULN, upper limit of normal.

Reductions (LS mean) in ALP from baseline at all time points through month 6 were greater in both seladelpar treatment groups versus the placebo group. At month 3, mean ALP decreased by 3.7% from baseline in the placebo group, whereas it decreased by 35.7% in the seladelpar 5-mg group and by 44.2% in the 10-mg group (*p* < 0.0001 for both groups) (Figure 5A, B). The reductions in ALP from baseline were significantly greater in the seladelpar 10-mg group versus the 5-mg group at months 1 and 3 (*p* ≤ 0.002 at both time points).

Reductions from baseline in ALT were also greater in the seladelpar groups versus the placebo group at all time points through month 6, with LS mean (median) reductions of 4.0% (3.0%), 23.4% (24.3%), and 16.7% (28.3%) observed in the placebo, 5-, and 10-mg groups, respectively, at month 3 (Figure 5C, D). In addition, among patients with ALT elevations at baseline, 18% (5/28), 52% (15/29), and 50% (14/28) of patients in the placebo, 5-, and 10-mg groups, respectively, achieved normalization at month 3. In contrast, among patients with normal baseline ALT levels, ALT levels rose above normal at month 3 in 7% (2/28; both 1.2×ULN), 0% (0/25), and 12% [3/25; 1.02×ULN, 1.29×ULN, and 1.88×ULN (this patient was < ULN at months 6 and 9)] of patients in the placebo, 5-, and 10-mg groups, respectively.

Compared with placebo, seladelpar was associated with greater improvements in other serum liver biochemical markers and in lipid levels through month 6. At month 3, LS mean reductions in total and indirect bilirubin were 6.1% and 6.5%, respectively, in the seladelpar 5-mg group and 4.0% and 6.8%, respectively, in the 10-mg group, while patients receiving placebo experienced a 0.9% increase in total bilirubin and a 1.1% increase in indirect bilirubin (Supplemental Figure S5A-B, S5E-F, http://links.lww.com/HEP/F475). At month 3, direct bilirubin levels decreased by an LS mean of 2.6% in the seladelpar 5-mg group and increased by an LS mean of 5.2% in the 10-mg group and 0.8% in the placebo group (Supplemental Figure S5C, D, http://links.lww.com/HEP/F475). LS mean reductions in both AST and GGT at month 3 were greater with seladelpar (8.5% and 30.3%, respectively, in the 5-mg group and 4.8% and 36.4%, respectively, in the 10-mg group) versus placebo (0.2% and 6.5%, respectively) (Supplemental Figure S5G–J, http://links.lww.com/HEP/F475). Compared with placebo, more than twice the proportion of patients treated with seladelpar (placebo, 17.1%; seladelpar 5 mg, 43.6%; and seladelpar 10 mg, 40.5%) shifted from a GGT level ≥ 3.2×ULN at baseline to < 3.2×ULN at month 3 (Supplemental Table S4, http://links.lww.com/HEP/F475). Only 1 patient (placebo group) shifted from below to above 3.2×ULN at baseline to month 3.

At month 3, seladelpar decreased mean total cholesterol, LDL-cholesterol (LDL-C), and triglyceride levels by 3.7%, 5.6%, and 5.9%, respectively, in the 5-mg group and by 4.4%, 8.2%, and 13.1%, respectively, in the 10-mg group compared with decreases of 1.8%, 0.6%, and 0.6%, respectively, in the placebo group (Supplemental Figure S5K–P, http://links.lww.com/HEP/F475). Seladelpar also increased mean HDL-cholesterol (HDL-C) levels from baseline at month 3 by 1.0% in the seladelpar 5-mg group and by 6.7% in the 10-mg group (Supplemental Figure S5Q, R, http://links.lww.com/HEP/F475). In contrast, mean HDL-C decreased 3.0% from baseline in the placebo group at month 3.

#### Bile acid synthesis (C4) and IgM concentrations

Seladelpar was also associated with greater LS mean reductions in serum levels of C4 and IgM at months 1 and 3 compared with the placebo group (Supplemental Table S5, http://links.lww.com/HEP/F475). At month 3, C4 levels decreased from baseline by an LS mean of 17.5% in the seladelpar 5-mg group and 24.8% in the 10-mg group but increased by an LS mean of 3.6% in the placebo group. C4 data were available in only 2 patients at month 6. IgM levels decreased by an LS mean of 10.2% in the seladelpar 5-mg group, 12.4% in the 10-mg group, and 5.7% in the placebo group at month 3. A similar pattern in reductions was observed at months 1 and 6.

### Safety

Mean (SD) duration of exposure was 17.7 (11.7) weeks in the total population. The large SD is due to the early termination of the study with patients having a wide range of treatment durations. Overall, the proportion of patients with ≥1 TEAE was greater in the placebo group (73.6%) versus either the seladelpar 5-mg (62.9%) or 10-mg (65.2%) groups (Table 2). The most common TEAEs overall were pruritus (qualitative reporting), upper abdominal pain, and nausea; the TEAE reported most often in the placebo group was pruritus. Serious TEAEs occurred in 3.4% of patients in both the placebo and seladelpar 5-mg groups and in 1.1% of patients in the seladelpar 10-mg group. Treatment-related TEAEs occurred in 21.1% of patients overall, the most frequently reported being pruritus (5.7% of patients in the placebo group and 2.2% and 3.4% in the seladelpar 5-mg and 10-mg groups, respectively) and nausea (2.3% of patients in the placebo group and 3.4% and 2.2% in the seladelpar 5-mg and 10-mg groups, respectively). All treatment-related TEAEs were grade 1 or 2. There were no grade 4 events or deaths in the study.

**TABLE 2:** Summary of safety events

	Placebo (N=87)	Seladelpar 5 mg (N=89)	Seladelpar 10 mg (N=89)	Total (N=265)
Duration of exposure (wk), mean (SD)	17.8 (11.2)	17.6 (12.1)	17.6 (12.0)	17.7 (11.7)
TEAE, n (%)				
≥1 TEAE	64 (73.6)	56 (62.9)	58 (65.2)	178 (67.2)
≥1 serious TEAE	3 (3.4)	3 (3.4)	1 (1.1)	7 (2.6)
≥1 TEAE Grade ≥ 3	6 (6.9)	3 (3.4)	5 (5.6)	14 (5.3)
≥1 TEAE leading to study drug discontinuation	2 (2.3)	2 (2.2)	2 (2.2)	6 (2.3)
≥1 treatment-related TEAE	16 (18.4)	25 (28.1)	15 (16.9)	56 (21.1)
≥1 serious treatment-related TEAE	0	0	0	0
≥1 treatment-related TEAE Grade ≥ 3	0	0	0	0
Deaths, n (%)	0	0	0	0
Common TEAEs (≥5% incidence in any treatment group), n (%)				
Pruritus (qualitative)	11 (12.6)	3 (3.4)	10 (11.2)	24 (9.1)
Abdominal pain upper	3 (3.4)	8 (9.0)	6 (6.7)	17 (6.4)
Nausea	4 (4.6)	5 (5.6)	7 (7.9)	16 (6.0)
Arthralgia	5 (5.7)	5 (5.6)	4 (4.5)	14 (5.3)
Fatigue	8 (9.2)	2 (2.2)	4 (4.5)	14 (5.3)
Headache	1 (1.1)	5 (5.6)	7 (7.9)	13 (4.9)
Upper respiratory tract infection	2 (2.3)	6 (6.7)	4 (4.5)	12 (4.5)
Constipation	2 (2.3)	5 (5.6)	3 (3.4)	10 (3.8)
Sinusitis	5 (5.7)	2 (2.2)	1 (1.1)	8 (3.0)
Urinary tract infection	0	2 (2.2)	5 (5.6)	7 (2.6)
Dry mouth	0	5 (5.6)	1 (1.1)	6 (2.3)

Abbreviations: n, number of patients in the category; N, number of patients in the treatment group; TEAE, treatment-emergent adverse event.

Six patients discontinued study drug due to TEAEs: 2 in the placebo group (increased bilirubin and atrioventricular block), 2 in the seladelpar 5-mg group (pruritus and adenoid cystic carcinoma), and 2 in the seladelpar 10-mg group (pruritus, insomnia, and rheumatoid arthritis). No grade 3 or greater elevations in serum aminotransferase activity were reported, and no muscle, renal, or pancreatic safety concerns were reported. The safety profile was comparable between patients with and without cirrhosis at baseline (Supplemental Table S6, http://links.lww.com/HEP/F475). Changes from baseline through month 6 in serum creatinine (renal) and creatine kinase (muscle) levels are summarized in Supplemental Table S7, http://links.lww.com/HEP/F475. A trend of minimal increases with seladelpar versus placebo was observed for both serum creatinine and creatine kinase, although mean levels for both remained within normal ranges in all treatment groups.

## DISCUSSION

New approved therapies remain important for people living with PBC. Current agents do not optimally address disease activity or symptoms. Seladelpar is a selective PPARδ agonist that has documented anticholestatic, anti-inflammatory, and antipruritic effects.^19,20^ This phase 3, randomized, placebo-controlled study evaluated the efficacy of adding seladelpar (5 or 10 mg) to UDCA or using seladelpar as monotherapy in patients with PBC at high risk for progression. As early as month 3 following treatment initiation, seladelpar caused rapid, dose-dependent, significant, and clinically meaningful improvements in validated serum markers of cholestasis and liver injury as well as pruritus. At month 3, 78.2% of patients in the seladelpar 10-mg group achieved the primary composite end point (ALP < 1.67×ULN, ≥15% ALP decrease, and total bilirubin ≤ ULN), which was significantly greater than the 12.5% response to placebo. Nearly 1 in 3 patients who received the 10-mg dose achieved ALP normalization compared with none in the placebo group. Seladelpar also significantly reduced serum ALP values in a dose-dependent manner through month 6. Associated biochemical markers of PBC disease activity, including ALT, AST, total bilirubin, GGT, and IgM, were dose-dependently reduced by seladelpar through month 6.

Among patients with clinically significant itch (baseline pruritus NRS ≥4), the pruritus NRS significantly decreased at month 3 with seladelpar 10 mg compared with placebo. The proportion of patients with a clinically meaningful reduction (≥2 points)^23^ in pruritus NRS was also significantly greater with seladelpar 10 mg versus placebo at month 3. These results were supported by the improvement observed in secondary instruments that assess pruritus, the 5-domain itch scale and the PBC-40 questionnaire itch domain. Improvement in pruritus has been previously observed with treatment with seladelpar 10 mg QD through 1 year.^20^ Pruritus is a significant burden for patients with PBC that is often undertreated,^28–30^ suggesting that seladelpar has the potential to add this benefit to that of improving biomarkers associated with disease progression. The PBC-40 total score, which sums diverse individual quality-of-life domain scores, did not improve when examined for the entire population at month 3. Documenting changes in the PBC-40 itch domain for all patients, including those with low levels of itch, has been previously noted as a challenge because of a floor effect.^31^ In our study, restricting the prespecified analysis to NRS ≥4 serves to overcome this effect. The improvement in pruritus with seladelpar contrasts with obeticholic acid, a bile acid analog and FXR agonist that is conditionally approved as a second-line treatment for PBC, which can induce or worsen pruritus in a dose-dependent manner.^15,32,33^

Seladelpar appeared safe and well tolerated, with no deaths or grade 3 or greater ALT or AST elevations. Pruritus (qualitative) was the most common adverse event; however, incidence was highest in the placebo group.

A variety of approaches are being pursued for new therapies in PBC. Direct comparisons are difficult because of study design differences. The 78.2% composite end point response rate at month 3 observed for seladelpar 10 mg in this study seems to be similar or better than those reported with other PBC therapies, including obeticholic acid,^15^ the PPARα/δ agonist in development, elafibranor,^34^ and the PPARα/γ agonist in development, saroglitazar.^35^ An important controlled clinical trial of the pan-PPAR agonist bezafibrate used a different end point of biochemical normalization with encouraging results.^36^

The anticholestatic, anti-inflammatory, and potentially antifibrotic properties of seladelpar are predicted to affect disease progression and improve outcomes. PPARδ is expressed in hepatocytes,^37^ where activation by seladelpar suppresses bile acid synthesis by reducing cholesterol 7 α-hydroxylase [*Cyp7a1*] expression through the FGF21 signaling pathway, independently of the nuclear bile acid receptor, FXR.^38^ C4, a downstream metabolite of CYP7A1, the rate-limiting enzyme for bile acid synthesis, is decreased with seladelpar, in keeping with the PPARδ-mediated downregulation of bile acid synthesis.^18,20,38^ PPARδ is also expressed in cholangiocytes,^39^ which use it to regulate transporters involved in the absorption and secretion of bile components, as confirmed in mouse liver studies showing that seladelpar regulates the cholesterol transporter ABCG5/ABCG8.^18^ Activation of PPARδ also induces anti-inflammatory effects in macrophages, including Kupffer cells,^40^ as confirmed in a study showing that seladelpar reduced macrophage numbers, fibrosis, and other markers of stellate cell activity in a mouse model.^41^

A limitation of this study is its short duration due to early termination. However, although the study was stopped early and the primary composite biochemical end point was amended to 3 months, more than three-fourths of patients achieved the end point. In addition, in a phase 2 study, seladelpar efficacy observed after 3 months of treatment was sustained through 1 year, including ALP reduction and achievement of ALP normalization and composite efficacy end point.^19^ Similarly, although the mean duration of exposure was only 17.7 weeks, phase 2 data suggest that seladelpar treatment is safe and well tolerated through 1 year. This study is also limited by the lack of data on liver stiffness and other markers of fibrosis in these patients. A strength of this study is that it used a prespecified hierarchical statistical methodology in which patients treated with seladelpar 10 mg achieved meaningful improvement in both disease activity (composite biochemical response and normalization of ALP) and pruritus end points.

In conclusion, in this placebo-controlled, randomized trial, the potent and selective PPARδ agonist seladelpar, at an optimal dose of 10 mg daily, provided clinically significant anticholestatic effects and reduced signs of liver injury and pruritus in patients with PBC. Treatment was not associated with emergent safety concerns. The efficacy and safety profile of seladelpar in this and previous studies suggests its potential use as second-line therapy to address disease activity and symptoms. A 52-week, phase 3, randomized, placebo-controlled, registration study to confirm seladelpar 10 mg QD efficacy and safety is ongoing (NCT04620733).

## Supplementary Material

SUPPLEMENTARY MATERIAL
