# Supplementary material for: Seladelpar efficacy and safety at 3 months in patients with primary biliary cholangitis: ENHANCE, a phase 3, randomized, placebo-controlled study
Source: Hepatology. 2023 Apr 6;78(2):397–415. doi: 10.1097/HEP.0000000000000395 (PMC10344437; doi:10.1097/HEP.0000000000000395)
Supplement: SUPPLEMENTARY MATERIAL [file hep-78-397-s001.pdf]

Seladelpar Efficacy and Safety at 3 Months in Patients With Primary Biliary Cholangitis: ENHANCE, a Phase 3, Randomized, Placebo-controlled Study

Short title: Seladelpar treatment for primary biliary cholangitis

Authors:

Gideon M. Hirschfield,[1] Mitchell L. Shiffman,[2] Aliya Gulamhusein,[3] Kris V. Kowdley,[4] John M. Vierling,[5] Cynthia Levy,[6] Andreas E. Kremer,[7] Ehud Zigmond,[8] Pietro Andreone,[9] Stuart C. Gordon,[10] Christopher L. Bowlus,[11] Eric J. Lawitz,[12] Richard J. Aspinall,[13] Daniel S. Pratt,[14] Karina Raikhelson,[15, 16] Maria Sarai Gonzalez-Huezo,[17] Michael A. Heneghan,[18] Sook-Hyang Jeong,[19] Alma L. Ladrón de Guevara,[20] Marlyn J. Mayo,[21] George N. Dalekos,[22] Joost P.H. Drenth,[23] Ewa Janczewska,[24] Barbara A. Leggett,[25] Frederik Nevens,[26] Victor Vargas,[27] Eli Zuckerman,[28] Christophe Corpechot,[29] Eduardo Fassio,[30] Holger Hinrichsen,[31] Pietro Invernizzi,[32,33] Palak J. Trivedi,[34-37] Lisa Forman,[38] David E.J. Jones,[39] Stephen D. Ryder,[40] Mark G. Swain,[41] Alexandra Steinberg,[42] Pol F. Boudes,[43] Yun-Jung Choi,[44] Charles A. McWherter,[45] ENHANCE Study Group*

Affiliations:

[1]Toronto Centre for Liver Disease, University Health Network and Division of Gastroenterology and Hepatology, University of Toronto, Toronto, Ontario, Canada; [2]Director, Liver Institute of Virginia, Bon Secours Mercy Health, Bon Secours Liver Institute of Richmond, Bon Secours Liver Institute of Hampton Roads, Richmond and Newport News, Virginia; [3]Hepatologist, Toronto Centre for Liver Disease, University Health Network and Department of Medicine, University of Toronto, Toronto, Ontario, Canada; [4]Director, Liver Institute Northwest, Seattle, Washington; [5]Chief of Hepatology and Professor, Departments of Medicine and Surgery, Baylor

College of Medicine, Houston, Texas; [6]Associate Director, Schiff Center for Liver Diseases, University of Miami Miller School of Medicine, Miami, Florida; [7]Clinician Scientist and Professor of Hepatology, Department of Gastroenterology and Hepatology, University Hospital Zürich, Zürich, Switzerland; [8]Director, Center for Autoimmune Liver Diseases, Tel-Aviv Sourasky Medical Center and Sackler Faculty of Medicine, Tel-Aviv University, Tel-Aviv, Israel; [9]Associate Professor, Division of Internal Medicine, Department of Medical and Surgical Sciences, Maternal-Infantile and Adult, University of Modena and Reggio Emilia, Modena, Italy; Chief, Postgraduate School of Allergy and Clinical Immunology, University of Modena and Reggio Emilia, Italy; [10]Director, Division of Hepatology, Henry Ford Hospital and Professor, Wayne State University School of Medicine, Detroit, Michigan; [11]Chief, Division of Gastroenterology and Hepatology, University of California Davis School of Medicine, Sacramento, California; [12]Vice President, Scientific and Research Development, The Texas Liver Institute; Clinical Professor of Medicine, University of Texas Health San Antonio, San Antonio, Texas; [13]Consultant, Department of Hepatology, Portsmouth Liver Centre, Portsmouth Hospitals National Health Service Trust, Queen Alexandra Hospital, Portsmouth, United Kingdom; [14]Director, Autoimmune and Cholestatic Liver Center, Massachusetts General Hospital, Boston, Massachusetts; [15]Professor, Saint Petersburg State University, St. Petersburg, Russia; [16]Doctor of Medical Sciences, City Hospital 31, St. Petersburg, Russia; [17]Head, Department of Gastroenterology, Instituto De Seguridad Social del Estado de México y Municipios, Toluca de Lerdo, Mexico; [18]Consultant Hepatologist and Clinical Director, King's College Hospital National Health Service Foundation Trust, London, United Kingdom; [19]Professor, Department of Internal Medicine, Seoul National University Bundang Hospital, Seongnam, Korea; [20]Director, Center of Research and Gastroenterology, Mexico City, Mexico;

[21]Professor of Internal Medicine, Division of Digestive and Liver Diseases, University of Texas Southwestern, Dallas, Texas; [22]Professor and Chairman, Department of Medicine and Research Laboratory of Internal Medicine, National Expertise Center of Greece in Autoimmune Liver Diseases, European Reference Network on Hepatological Diseases (ERN RARE-LIVER), General University Hospital of Larissa, Larissa, Greece; [23]Professor of Gastroenterology and Hepatology, Radboudumc, Nijmegen, Netherlands; [24]Adjuct Professor, Department of Basic Medical Sciences, Faculty of Health Sciences in Bytom, Medical University of Silesia, Katowice, Poland; Head, ID Clinic, Myslowice, Poland; [25]Professor of Medicine, School of Medicine, University of Queensland, Herston, Queensland, Australia; [26]Professor of Medicine, Hepatology and Liver Transplantation, University Hospitals KU Leuven, Belgium; Center of European Reference Network (ERN) RARE-LIVER, Leuven, Belgium; [27]Senior Consultant, Professor of Medicine, Liver Unit, Hospital Universitari Vall d'Hebron, Universitat Autònoma de Barcelona; Centro de Investigación Biomédica en Red de Enfermedades Hepáticas y Digestivas (CIBEREHD), Barcelona, Spain; [28]Director, Liver Unit, Carmel Medical Center, Technion, Faculty of Medicine, Israeli Association for the Study of the Liver, Haifa, Israel; [29]Head, Centre de Référence Maladies Rares site Coordonnateur, Maladies Inflammatoires des Voies Biliaires et Hépatites Auto-immunes (MIVB-H), Filière Maladies Rares: Maladies Rares du Foie de l'Adulte et de l'Enfant (FILFOIE), European Reference Network (ERN) RARE-LIVER, Inserm, Centre de Recherche Saint-Antoine (CRSA), Hôpital Saint-Antoine (APHP) et Sorbonne Universités, Paris, France; [30]DIM Clínica Privada, Ramos Mejía, Buenos Aires province, Argentina; [31]Director, Gastroenterology–Hepatology Center Kiel, Kiel, Germany; [32]Dean, Center for Autoimmune Liver Diseases, Department of Medicine and Surgery, University of Milano-Bicocca, Monza, Italy; [33]Professor and Head, Division of

Gastroenterology, Fondazione IRCCS San Gerardo dei Tintori & European Reference Network on Hepatological Diseases (ERN RARE-LIVER), Monza, Italy; [34]Associate Professor and Honorary Consultant Hepatologist, National Institute for Health Research (NIHR), Birmingham Biomedical Research Centre, Centre for Liver and Gastroenterology Research, University of Birmingham, United Kingdom; [35]Liver Unit, University Hospitals Birmingham Queen Elizabeth, Birmingham, United Kingdom; [36]Institute of Immunology and Immunotherapy, University of Birmingham, United Kingdom; [37]Institute of Applied Health Research, University of Birmingham, United Kingdom; [38]Professor of Medicine, University of Colorado, Aurora, Colorado; [39]Professor, Institute of Cellular Medicine and National Institute for Health Research (NIHR) Newcastle Biomedical Research Centre, Newcastle University, Newcastle upon Tyne, United Kingdom; [40]Clinical Director, National Institute for Health Research (NIHR) Nottingham Biomedical Research Centre at Nottingham University Hospitals National Health Service (NHS) Trust and the University of Nottingham, Queens Medical Centre, Nottingham, United Kingdom; [41]Professor, Department of Medicine, University of Calgary, Calgary, Alberta, Canada; [42]Senior Medical Director, CymaBay Therapeutics, Newark, California; [43]Chief Medical Officer, CymaBay Therapeutics, Newark, California; [44]Senior Director of Clinical Science, CymaBay Therapeutics, Newark, California; [45]Chief Scientific Officer, CymaBay Therapeutics, Newark, California

*Names and affiliations are listed in Acknowledgements.

ENHANCE Study Group

Raul Adrover,[1] Saurabh Agrawal,[2] Elmar Aigner[3] Agustin Albillos Martinez,[4] Abu-Mouch Saif Alden,[5] Raul Jesús Andrade Bellido,[6] Marco Antonio Arrese Jiménez,[7] Walid Ayoub,[8] Seth J. Baum,[9] Ziv Ben-Ari,[10] Marina Berenguer,[11] Christoph Berg,[12] Fernando Oscar Bessone,[13] Alan

Bonder,[14] Brian B. Borg,[15] Carlos Gustavo Bresky Ruiz,[16] Peter Buggisch,[17] Jose Luis Calleja Panero,[18] Elizabeth J. Carey,[19] Michal Carmiel-Haggai,[20] Francesca Carubbi,[21] Pilar Castillo Grau,[22] Chin Lye Ch'ng,[23] Nicoleta-Claudia Cimpoeru,[24] Raúl Contreras Omaña,[25] Lynsey Corless,[26] Charlotte Costentin,[27] Marc Deschênes,[28] Yvonne Dörffel,[29] Predrag Dugalic,[30] Geoffrey Charles Farrell,[31] José Luis Fernández,[32] Annarosa Floreani,[33] Sven Francque,[34] Bradley L. Freilich,[35] Francisco Alejandro FusterSaldias,[36] Michael R. Galambos,[37] Joseph Galati,[38] Andrea Galli,[39] Nathalie Ganne-Carrie,[40] Natalia Geyvandova,[41] Liliana-Simona Gheorghe,[42] Richard Gilroy,[43] Aparna Goel,[44] Tobias Goeser,[45] Susan Greenbloom,[46] Waldemar Halota,[47] Stephen A. Harrison,[48] Marek Hartleb,[49] Jeong Heo,[50] Harald Hofer,[51] Gábor Horváth,[52] Jonathan C. Huang,[53] Jason Lee Huffman,[54] Béla Hunyady,[55] Steven Johnson,[56] Yiannis Kallis,[57] Arun Khazanchi,[58] Kyung-Ah Kim,[59] Seung Up Kim,[60] Yoon Jun Kim,[61] Anita Kohli,[62] Nicholas Kontorinis,[63] John R. Lake,[64] Kwan Sik Lee,[65] Tomasz Mach,[66] Richard Manch,[67] Yaakov Maor-Kendler,[68] Radu-Bogdan Mateescu,[69] Gerald Minuk,[70] Apurva A. Modi,[71] Martin William Moehlen,[72] Cristina Montón Rodríguez,[73] Rosa Maria Morillas Cunill,[74] Ioannis Mouzas,[75] Andrew Muir,[76] István Nagy,[77] Jing Hieng (Jeffrey) Ngu,[78] Joseph Odin,[79] Pavel Ogurtsov,[80] Pawel Pabjan,[81] Mangesh Pagadala,[82] Mária Papp,[83] Albert Parés,[84] Andrey Peskov,[85] Adam Peyton,[86] John Phillips,[87] Michael Porayko,[88] Anthony Post,[89] David C. Pound,[90] Mordechai Rabinovitz,[91] Kevin Rank,[92] K. Gautham Reddy,[93] Jaroslaw Regula,[94] Thomas Riley,[95] Manuel Romero Gómez,[96] Rifaat Safadi,[97] David Anthony Sheridan,[98] Marcelo Oscar Silva,[99] Marina G. Silveira,[100] Siddarth Sood,[101] Ioan Sporea,[102] Carmen Stanca,[103] Rudolf Stauber,[104] Petar Svorcan,[105] Won Young Tak,[106] Ryan M Taylor,[107] Douglas Thorburn,[108] Hillel Tobias,[109] Arnany Tourky Zekry,[110] Michael Trauner,[111] Christos Triantos,[112] Ella Veitsman,[113] Alexander James VennThompson,[114] Xavier Verhelst,[115] Elizabeth C. Verna,[116] Manfred von der

Ohe,[117] Frank Weilert,[118] Johannes Wiegand,[119] Kidist K. Yimam,[120] Seung Kew Yoon,[121] Ziad Younes,[122] Adam Seth Zivony,[123] Massimo Zuin[124]

ENHANCE Study Group Affiliations

[1]Centro de Hepatologia, La Plata, Buenos Aires, Argentina; [2]Tampa General Hospital, Tampa, Florida; [3]Salzburger LandesklinikenInnere Medzin I, Salzburg, Austria; [4]Gastroenterology and Hepatology, Hospital Universitario Ramon y Cajal, Madrid, Spain; [5]Hillel Yaffe Medical Center Liver Clinic, Hadera, Israel; [6]Hospital Universitario Virgen de la Victoria, Málaga, Spain; [7]Centro de Investigaciones Clínicas de La Pontificia Universidad, Católica de Chile (CICUC), Santiago, Región Metropolitana, Chile; [8]Cedars Sinai Medical Center, Los Angeles, California; [9]Excel Medical Clinical Trials, LLC, Boca Raton, Florida; [10]Liver Disease Center Sheba Medical Center, Ramat Gan, Israel; [11]Hospital Universitari i Politecnic La Fe de Valencia, Servicio Digestivo, Valencia, Spain; [12]University Hospital Tübingen-Innere Medizin I, Tübingen, Baden-Württemberg, Germany; [13]Hospital Provincial def Centenario, Rosario, Santa Fé, Argentina; [14]Beth Israel Deaconess Medical Center, Boston, Massachusetts; [15]Southern Therapy and Advanced Research (STAR), LLC, Jackson, Mississippi; [16]Centro Clinico Mediterráneo, La Serena, Coquimbo, Chile; [17]Ifi - Studien und Projekte GmbH Haus L, Hamburg, Germany; [18]Gastroenterología y Hepatología, Hospital Universitario Puerta de Hierro–Majadahonda, Madrid, Spain; [19]Mayo Clinic Arizona–Mayo Clinic Hospital, Phoenix, Arizona; [20]The Galilee Medical Center Liver Unit, Nahariya, Israel; [21]UO Complessa Medicina Metabolica, Ospedale Civile S. Agostino Estense (OCSAE), Modena, Italy; [22]Hospital Universitario La Paz, Madrid, Spain; [23]Singleton Hospital, Swansea, United Kingdom; [24]S.C. Sana Monitoring S.R.L., Bucharest, Romania; [25]Centro de Diabetes y Obesidad GRABER, Pachuca, Hidalgo, Mexico; [26]Hull and East Yorkshire Hospitals National Health Service (NHS) Trust, Hull, United

Kingdom; [27]CHU Grenoble Alpes-Service d'Hépato-Gastroentérologie, Grenoble, France ; [28]Chronic Viral Illness Service McGill University Health Centre (MUHC)/Royal Victoria, Montreal, Quebec City, Canada; [29]Charité-Universitätsmedizin Berlin, Berlin, Germany; [30]Clinical Hospital Center Zemun, Belgrade, Serbia; [31]Canberra Hospital, Garran, Australia; [32]Sanatorio Güemes, Buenos Aires, Argentina [33]Azienda Ospedaliera di Padova, U.O.C. Gastroenterologia, Padova, Italy; [34]University Hospital Antwerp (UZA), Edegem, Belgium; [35]Kansas City Research Institute, Kansas City, Missouri; [36]Centro de Investigaciones Clínicas Viña del Mar, Jardín Del Mar, Reñaca, Viña del Mar, Valparaíso, Chile; [37]Digestive Healthcare of Georgia, Atlanta, Georgia; [38]American Research Corporation, Houston, Texas; [39]Azienda Ospedalicro Universitaria Careggi Gastroenterologia Clinica, Firenze, Italy; [40]Hôpital Jean Verdier - Service d'Hépatologie, Bondy, France ; [41]SBHI of Stavropol region "Stavropol Regional Clinical Hospital", Stavropol, Russia ; [42]Fundeni Clinical Institute, Bucharest, Romania ; [43]Intermountain Medical Center - Transplant Services, Murray, Utah ; [44]Stanford University Medical Center, Redwood City, California; [45]Universitätsklinikum Köln Gastroenterologie und Hepatologie, Koeln, Germany; [46]Toronto Digestive Disease Associates, Inc., Vaughan, Ontario, Canada; [47]Wojewodzki Szpital Obserwacyjno-Zakazny im. Tadeusza Browicza, Bydgoszcz, Kujawsko-pomorskie, Poland; [48]Pinnacle Clinical Research, San Antonio, Texas; [49]Samodzielny Publiczny Szpital Kliniczny Nr 7 Slaskiego Uniwersytetu Medycznego w Katowicach, Katowice, Slaskie, Poland; [50]Pusan National University Hospital , Busan, Korea; [51]Klinikum Wels-Griekirchen GmbH Abteilung für Innere Medzin I, Wels, Austria; [52]Budai Hepatológiai Centrum, Budapest, Hungary; [53]University of Rochester Medical Center, Rochester, New York; [54]GIA Clinical Trials, LLC, Knoxville, Tennessee; [55]Somogy Megyei Kaposi Mór Oktató Kórház, Kaposvár, Hungary; [56]Southern DHB, Dunedin Hospital Dunedin,

New Zealand; [57]Barts Health National Health Service (NHS) Trust, Royal London Hospital, London, London, United Kingdom; [58]Florida Research Institute, Lakewood Ranch, Florida; [59]Inje University Ilsan Paik Hospital, Goyang-si, Gyeonggi-do, Korea; [60]Severance Hospital, Yonsei University Health System, Seoul, Korea; [61]Seoul National University Hospital, Seoul, Korea; [62]Arizona Liver Health, Chandler, Arizona; [63]Royal Perth Hospital Perth, Western Australia, Australia; [64]University of Minnesota, Phillips-Wangensteen Building, Minneapolis, Minnesota; [65]Gangnam Severance Hospital, Yonsei University Health System, Seoul, Korea; [66]SP ZOZ Szpital Uniwersytecki w Krakowie, Oddzial Kliniczny Gastroenterologii i Hepatologii oraz Chorob Zakaznych, Krakow, Malopolskie, Poland; [67]Arizona Liver Health, Tucson, Arizona; [68]Kaplan Medical Center Liver Service, Rehovot, Israel; [69]Colentina Clinical Hospital, Bucharest, Romania; [70]University of Manitoba/John Buhler Research Centre/Health Sciences Centre, Winnipeg, Canada; [71]Baylor Scott and White Liver Consultants of Texas, Fort Worth, Texas; [72]Tulane University Health Sciences Center, New Orleans, Louisiana; [73]Hospital Clinico Universitario de Valencia, Medicina Digestiva, Valencia, Spain; [74]Hospital Universitario Germans Trias i Pujol, Badalona, Spain; [75]University General Hospital of Heraklion Voutes, Heraklion, Greece; [76]Duke University Medical Center, Durham, North Carolina; [77]Szegedi Tudományegyetem Szent-Györgyi Albert Klinikai Központ, I. Belgyógyászati Klinika Szeged, Hungary; [78]Christchurch Hospital, Christchurch, New Zealand; [79]Recanati/Miller Transplantation Institute, New York, New York; [80]FSAEI of HE "Peoples' Friendship University of Russia (RUDN)", RUDN Liver Centre, Moscow, Russia; [81]Wojewodzki Szpital Zespolony, Swietokrzyskie, Poland; [82]The Liver Institute at Methodist Dallas Medical Center, Dallas, Texas; [83]Debreceni Egyetem Klinikai Központ, Debrecen, Hungary; [84]Hospital Clinic de Barcelona, Barcelona, Spain; [85]State Healthcare Institution «Ulyanovsk Regional Clinical Hospital»,

Ulyanovsk, Russia; [86]Northeast Clinical Research Center, LLC, Bethlehem, Pennsylvania; [87]Digestive Health Specialist, PA, Tupelo, Mississippi; [88]Vanderbilt University Medical Center - Digestive Disease Center, Nashville, Tennessee; [89]University Hospitals Cleveland Medical Center, Cleveland, Ohio; [90]Indianapolis Gastroenterology Research Foundation, Indianapolis, Indiana; [91]University of Pittsburgh, Pittsburgh, Pennsylvania; [92]MNGI Digestive Health—Northeast Minneapolis Clinic, Minneapolis, Minnesota; [93]University of Chicago, Chicago, Illinois; [94]Centrum Onkologii Instytut im. Marii Sklodowskiej-Curie Klinika Gastroenterologii, Warszawa, Mazowieckie, Poland; [95]Penn State Health Milton S. Hershey Medical Center, Hershey, Pennsylvania; [96]Hospital Universitario Virgen del Rocio, Sevilla, Spain; [97]Hadassah Medical Center Liver Unit, Jerusalem, Israel; [98]Plymouth Hospitals National Health Service (NHS) Trust, Devon, United Kingdom; [99]Hospital Universitario Austral, Pilar, Buenos Aires, Argentina; [100]Yale School of Medicine, Digestive Diseases, Internal Medicine, New Haven, Connecticut; [101]The Royal Melbourne Hospital, Parkville, Victoria, Australia; [102]"Pius Brînzeu" Emergency Clinical County Hospital, Timisoara, Romania; [103]NYU Langone Health, New York, New York; [104]Medizinische Universität Graz Universitätsklinikum für Innere Medzin Klinische Abteilung für Gastroenterologie und Hepatologie, Graz, Austria; [105]Clinical Hospital Center Zvezdara, Belgrade, Serbia; [106]Kyungpook National University Hospital, Daegu, Korea; [107]University of Kansas Medical Center, Kansas City, Kansas; [108]Royal Free London National Health Service (NHS) Foundation Trust Royal Free Hospital, London, United Kingdom; [109]Manhattan Clinical Research, LLC, New York, New York; [110]St. George Hospital, Kogarah, New South Wales, Australia; [111]Medizinische Universität Wien Universitätsklinik für Innere Medzin III Klinische Abteilung für Gastroenterologie und Hepatologie, Wien, Austria; [112]University General Hospital of Patras Rion, Patras, Greece; [113]Rambam Medical Center Liver

Disease Center, Haifa, Israel; [114]St. Vincent's Hospital Melbourne Limited, Fitzroy, Victoria, Australia; [115]UZ Gent, Gent, Belgium; [116]Center for Liver Disease and Transplantation, Columbia University Medical Center, New York, New York; [117]Gastroenterologische Gemeinschaftspraxis Herne, Nordrhein-Westfalen, Germany; [118]Waikato Hospital, Menzies Building, Hamilton, New Zealand; [119]Universitätsklinikum Leipzig, Klinik und Poliklinik für Gastroenterologie und Rheumatologie, Leipzig, Germany; [120]Sutter Pacific Medical Foundation - California Pacific Medical Center, San Francisco, California; [121]The Catholic University of Korea, Seoul Street Mary's Hospital, Seoul, Korea; [122]Gastro One, Germantown, Tennessee; [123]Asheville Gastroenterology Associates, a Division of Digestive Health Partners, PA, Asheville, North Carolina; [124]U.O.C. Medicina Generate VI - Epatologia e Gastroenterologia, Milan, Italy

Corresponding Author:

Gideon M. Hirschfield

Toronto General Hospital, Toronto Centre for Liver Disease

9th Floor Eaton Building, North Wing 219-B

200 Elizabeth Street, Toronto, Ontario M5G 2C4, Canada

Tel: 416-340-4548

Fax: 416-340-4533

Email: gideon.hirschfield@uhn.ca

SUPPLEMENTAL INFORMATION

Supplemental Tables

Supplemental Table S1. PBC response criteria

	Placebo	Seladelpar 5mg	Seladelpar 10mg
**Rotterdam criteria[a]**			
Month 1, n/N (%)	2/78 (3)	3/80 (4)	6/79 (8)
95% CI	0.3, 9.0	0.8, 10.6	2.8, 15.8
Month 3, n/N (%)	0/56 (0)	1/56 (2)	1/55 (2)
95% CI	0, 6.4	0, 9.6	0, 9.7
Month 6, n/N (%)	0/23 (0)	2/26 (8)	1/20 (5)
95% CI	0, 14.8	0.9, 25.1	0.1, 24.9
**Barcelona criteria[b]**			
Month 1, n/N (%)	0/78 (0)	12/80 (15)	39/79 (49)
95% CI	0, 4.6	8.0, 24.7	37.9, 60.9
Month 3, n/N (%)	0/56 (0)	21/56 (38)	36/55 (66)
95% CI	0, 6.4	24.9, 51.5	51.4, 77.8
Month 6, n/N (%)	1/23 (4)	10/26 (39)	14/20 (70)
95% CI	0.1, 21.9	20.2, 59.4	45.7, 88.1
**Paris I & II criteria**			
**Paris I criteria[c]**			
Month 1, n/N (%)	52/78 (67)	60/80 (75)	67/79 (85)
95% CI	55.1, 76.9	64.1, 84.0	75.0, 91.9

	Placebo	Seladelpar 5mg	Seladelpar 10mg
Month 3, n/N (%)	36/56 (64)	45/56 (80)	48/55 (87)
95% CI	50.4, 76.6	67.6, 89.8	75.5, 94.7
Month 6, n/N (%)	17/23 (74)	19/26 (73)	17/20 (85)
95% CI	51.6, 89.8	52.2, 88.4	62.1, 96.8
**Paris II criteria**[d]			
Month 1, n/N (%)	8/78 (10)	31/80 (39)	40/79 (51)
95% CI	4.5, 19.2	28.1, 50.3	39.1, 62.1
Month 3, n/N (%)	5/56 (9)	28/56 (50)	32/55 (58)
95% CI	3.0, 19.6	36.3, 63.7	44.1, 71.3
Month 6, n/N (%)	3/23 (13)	14/26 (54)	12/20 (60)
95% CI	2.8, 33.6	33.4, 73.4	36.1, 80.9
**Toronto I criteria**[e]			
Month 1, n/N (%)	11/78 (14)	43/80 (54)	54/79 (68)
95% CI	7.3, 23.8	42.2, 65.0	56.9, 78.4
Month 3, n/N (%)	10/56 (18)	36/56 (64)	45/55 (82)
95% CI	8.9, 30.4	50.4, 76.6	69.1, 90.9
Month 6, n/N (%)	8/23 (35)	16/26 (62)	15/20 (75)
95% CI	16.4, 57.3	40.6, 79.8	50.9, 91.3
**Toronto II criteria**[f]			
Month 1, n/N (%)	16/78 (21)	47/80 (59)	59/79 (75)
95% CI	12.2, 31.2	47.2, 69.6	63.6, 83.8

		Seladelpar	
	Placebo	5mg	10mg
Month 3, n/N (%)	14/56 (25)	40/56 (71)	45/55 (82)
95% CI	14.4, 38.4	57.8, 82.7	69.1, 90.9
Month 6, n/N (%)	9/23 (39)	17/26 (65)	16/20 (80)
95% CI	19.7, 61.5	44.3, 82.8	56.3, 94.3

Two-sided 95% exact (Clopper-Pearson) CIs are provided.

[a]Normalization of abnormal total bilirubin and/or albumin

[b]ALP ≤1×ULN or >40% decrease

[c]ALP <3×ULN, AST <2×ULN, and total bilirubin ≤1mg/dL

[d]ALP <1.5×ULN, AST <1.5×ULN, and total bilirubin ≤1mg/dL

[e]ALP ≤1.67×ULN

[f]ALP ≤1.76×ULN

Abbreviations: ALP, alkaline phosphatase; AST, aspartate aminotransferase; CI, confidence interval; N, number of patients in the treatment group; n, number of patients in the category; ULN, upper limit of normal.

Supplemental Table S2. Proportion of patients who achieved individual composite endpoint components or ALP <1.5 × ULN through Month 6

		Seladelpar	
	Placebo	5mg	10mg
Month 1, N	78	80	79
ALP <1.67×ULN, n (%)	11 (14)	43 (54)	54 (68)
≥15% decrease in ALP, n (%)	12 (15)	74 (93)	76 (96)
Total bilirubin ≤ULN, n (%)	71 (91)	68 (85)	75 (95)
ALP <1.5×ULN, n (%)	8 (10)	37 (46)	47 (60)
Month 3, N	56	56	55
ALP <1.67×ULN, n (%)	10 (18)	36 (64)	45 (82)
≥15% decrease in ALP, n (%)	13 (23)	53 (95)	52 (95)
Total bilirubin ≤ULN, n (%)	51 (91)	48 (86)	51 (93)
ALP <1.5×ULN, n (%)	5 (9)	33 (59)	41 (75)
Month 6, N	23	26	20
ALP <1.67×ULN, n (%)	8 (35)	16 (62)	15 (75)
≥15% decrease in ALP, n (%)	7 (30)	22 (85)	17 (85)
Total bilirubin ≤ULN, n (%)	20 (87)	23 (89)	17 (85)
ALP <1.5×ULN, n (%)	3 (13)	15 (58)	13 (65)

Patients who discontinued treatment prior to time point due to reasons other than study termination and who did not have evaluable data at time point were considered nonresponders. Abbreviations: ALP, alkaline phosphatase; N, number of patients in the treatment group; n, number of patients in the category; ULN, upper limit of normal.

Supplemental Table S3. Proportions of patients with pruritus NRS scores ≥4 or <4 at baseline and post baseline

Post Baseline Pruritus NRS Score		<4			≥4		
**Baseline Pruritus NRS Score**	**Time point**	**Placebo**	**5mg**	**10mg**	**Placebo**	**5mg**	**10mg**
≥4	**Month 1**	19.2 (5/26)	23.1 (6/26)	48.1 (13/27)	80.8 (21/26)	76.9 (20/26)	51.9 (14/27)
	**Month 3**	27.8 (5/18)	47.1 (8/17)	61.1 (11/18)	72.2 (13/18)	52.9 (9/17)	38.9 (7/18)
	**Month 6**	66.7 (4/6)	55.6 (5/9)	100 (7/7)	33.3 (2/6)	44.4 (4/9)	0 (0/7)
<4	**Month 1**	93.3 (56/60)	98.4 (60/61)	93.5 (58/62)	6.7 (4/60)	1.6 (1/61)	6.5 (4/62)
	**Month 3**	97.5 (39/40)	97.6 (40/41)	92.7 (38/41)	2.5 (1/40)	2.4 (1/41)	7.3 (3/41)
	**Month 6**	95.2 (20/21)	94.7 (18/19)	95.2 (20/21)	4.8 (1/21)	5.3 (1/19)	4.8 (1/21)

Data are % (n/N). Shaded cells indicate no shift from baseline; unshaded cells indicate a shift from baseline.

Abbreviations: N, number of patients in the treatment group; n, number of patients in the category; NRS, numerical rating scale.

Supplemental Table S4. Proportions of patients with GGT levels ≥3.2×ULN or <3.2×ULN at baseline and post baseline

Post Baseline GGT Level		<3.2×ULN			≥3.2×ULN		
**Baseline GGT Level**	**Time point**	**Placebo**	**5mg**	**10mg**	**Placebo**	**5mg**	**10mg**
**≥3.2×ULN**	**Month 1**	13.2 (7/53)	31.5 (17/54)	36.4 (20/55)	86.8 (46/53)	68.5 (37/54)	63.6 (35/55)
	**Month 3**	17.1 (6/35)	43.6 (17/39)	40.5 (15/37)	82.9 (29/35)	56.4 (22/39)	59.5 (22/37)
**<3.2×ULN**	**Month 1**	100 (25/25)	100 (24/24)	100 (23/23)	0 (0/25)	0 (0/24)	0 (0/23)
	**Month 3**	95.2 (20/21)	100 (15/15)	100 (16/16)	4.8 (1/21)	0 (0/15)	0 (0/16)

Data are % (n/N). Shaded cells indicate no shift from baseline; unshaded cells indicate a shift from baseline.

Abbreviations: GGT, gamma glutamyl transferase; N, number of patients in the treatment group; n, number of patients in the category; ULN, upper limit of normal.

Supplemental Table S5. Mean absolute values and LS mean relative (percent) change from baseline in 7α-hydroxy-4-cholesten-3-one (C4) and immunoglobulin M (IgM) through Month 6

		Seladelpar	
	Placebo	5mg	10mg
**C4**			
Baseline, N	87	89	89
Mean (SD), ng/mL	27.3 (25.0)	23.1 (23.6)	20.7 (18.9)
Month 1, N	22	27	19
Mean (SD), ng/mL	24.1 (17.7)	19.6 (16.7)	16.1 (12.2)
LS mean (SE) change from baseline, %	21.3 (18.0)	6.9 (15.4)	-25.0 (18.1)
Month 3, N	55	52	51
Mean (SD), ng/mL	25.8 (20.5)	17.8 (15.6)	19.0 (27.2)
LS mean (SE) change from baseline, %	3.6 (9.6)	-17.5 (9.8)	-24.8 (10.0)
Month 6, N	0	1	1
Mean (SD), ng/mL	--	10.2 (--)	21.2 (--)
Mean (SD) change from baseline, %	--	4.6 (--)	-20.4 (--)

**IgM**			
Baseline, N	87	88	89
Mean (SD), mg/dL	357.9 (264.2)	331.6 (201.8)	316.5 (181.3)
Month 1, N	78	77	78
Mean (SD), mg/dL	341.7 (227.0)	314.5 (189.6)	290.7 (168.1)
LS mean (SE) change from baseline, %	-2.6 (1.4)	-5.7 (1.4)	-7.3 (1.4)
Month 3, N	55	53	53
Mean (SD), mg/dL	312.1 (221.7)	305.1 (196.5)	267.1 (151.2)
LS mean (SE) change from baseline, %	-5.7 (2.1)	-10.2 (2.1)	-12.4 (2.1)
Month 6, N	23	24	18
Mean (SD), mg/dL	314.7 (261.2)	301.4 (243.6)	301.1 (229.8)
LS mean (SE) change from baseline, %	-3.4 (4.8)	-8.7 (4.4)	-12.2 (4.6)

Abbreviations: C4, 7α-hydroxy-4-cholesten-3-one; IgM, immunoglobulin M; LS, least squares; N, number of patients in the treatment group; n, number of patients in the category; SD, standard deviation; SE, standard error.

Supplemental Table S6. Summary of treatment-emergent adverse events in patients with and without cirrhosis at baseline

	Patients With Cirrhosis			Patients Without Cirrhosis		
		Seladelpar			Seladelpar	
	Placebo (N=7)	5 mg (N=9)	10 mg (N=13)	Placebo (N=80)	5 mg (N=80)	10 mg (N=76)
**TEAE, n (%)**						
**≥1 TEAE**	7 (100)	5 (56)	8 (62)	57 (71)	51 (64)	50 (66)
**≥1 serious TEAE**	0	0	0	3 (4)	3 (4)	1 (1)
**≥1 Grade ≥3 TEAE**	0	0	0	6 (8)	3 (4)	5 (7)
**≥1 TEAE leading to study drug discontinuation**	1 (14)	0	1 (8)	1 (1)	2 (3)	1 (1)
**≥1 treatment-related TEAE**	4 (57)	2 (22)	1 (8)	12 (15)	23 (29)	14 (18)
**≥1 serious treatment-related TEAE**	0	0	0	0	0	0
**≥1 treatment-related TEAE Grade ≥3**	0	0	0	0	0	0
**Deaths**	0	0	0	0	0	0

	Patients With Cirrhosis			Patients Without Cirrhosis		
		Seladelpar			Seladelpar	
	**Placebo (N=7)**	**5 mg (N=9)**	**10 mg (N=13)**	**Placebo (N=80)**	**5 mg (N=80)**	**10 mg (N=76)**
**TEAEs occurring in ≥2 patients with cirrhosis in any treatment cohort**						
**Arthralgia**	1 (14)	1 (11)	2 (15)	4 (5)	4 (5)	2 (3)
**Fatigue**	2 (29)	0	2 (15)	6 (8)	2 (3)	2 (3)
**Pruritus (qualitative)**	1 (14)	0	2 (15)	10 (13)	3 (4)	8 (11)
**Abdominal pain upper**	0	1 (11)	2 (15)	3 (4)	7 (9)	4 (5)
**Headache**	0	0	3 (23)	1 (1)	5 (6)	4 (5)
**Dry eye**	0	1 (11)	2 (15)	2 (3)	2 (3)	1 (1)
**Nausea**	0	0	2 (15)	4 (5)	5 (6)	5 (7)
**Vomiting**	0	0	2 (15)	3 (4)	0	1 (1)
**Musculoskeletal chest pain**	0	0	2 (15)	0	0	1 (1)

All values are n (%) for patients in the safety population.

Adverse events were coded using MedDRA® version 21.0. MedDRA, Medical Dictionary for Regulatory Activities; n, number in category; N, number in treatment cohort; TEAE, treatment-emergent adverse event.

Supplemental Table S7. Mean absolute values and mean relative (percent) change from baseline in serum creatinine and creatine kinase through Month 6

		Seladelpar	
	**Placebo**	**5mg**	**10mg**
**Serum creatinine**			
Baseline, N	87	89	89
Mean (SD), mg/dL	0.71 (0.12)	0.70 (0.15)	0.72 (0.14)
Month 1, N	78	78	78
Mean (SD), mg/dL	0.73 (0.12)	0.73 (0.16)	0.76 (0.15)
Mean (SD) change from baseline, %	2.7 (7.9)	4.4 (10.5)	6.0 (10.8)
Month 3, N	56	54	53
Mean (SD), mg/dL	0.70 (0.11)	0.74 (0.16)	0.79 (0.16)
Mean (SD) change from baseline, %	4.0 (12.3)	5.0 (10.0)	7.2 (10.8)
Month 6, N	23	24	18
Mean (SD), mg/dL	0.68 (0.12)	0.81 (0.16)	0.84 (0.17)
Mean (SD) change from baseline, %	9.1 (10.3)	13.4 (13.3)	12.3 (10.2)

**Creatine kinase**			
Baseline, N	87	89	89
Mean (SD), U/L	70.8 (34.9)	78.3 (41.1)	80.3 (42.8)
Month 1, N	78	78	78
Mean (SD), U/L	81.7 (53.5)	81.4 (54.8)	92.1 (78.4)
Mean (SD) change from baseline, %	14.2 (55.8)	4.8 (50.7)	20.5 (78.5)
Month 3, N	56	54	53
Mean (SD), U/L	69.8 (32.2)	94.0 (104.4)	84.8 (46.1)
Mean (SD) change from baseline, %	5.4 (29.9)	8.1 (50.4)	10.8 (36.7)
Month 6, N	23	24	18
Mean (SD), U/L	75.3 (39.2)	67.0 (31.6)	75.1 (33.9)
Mean (SD) change from baseline, %	2.0 (23.7)	-1.3 (21.3)	17.2 (59.2)

Serum creatinine ULN for females is 1.2mg/dL. Creatine kinase ULN is 210U/L.

Abbreviations: LS, least squares; N, number of patients in the treatment group; n, number of patients in the category; SD, standard deviation.

Supplemental Figures

Supplemental Figure S1. Composite endpoint through Month 6

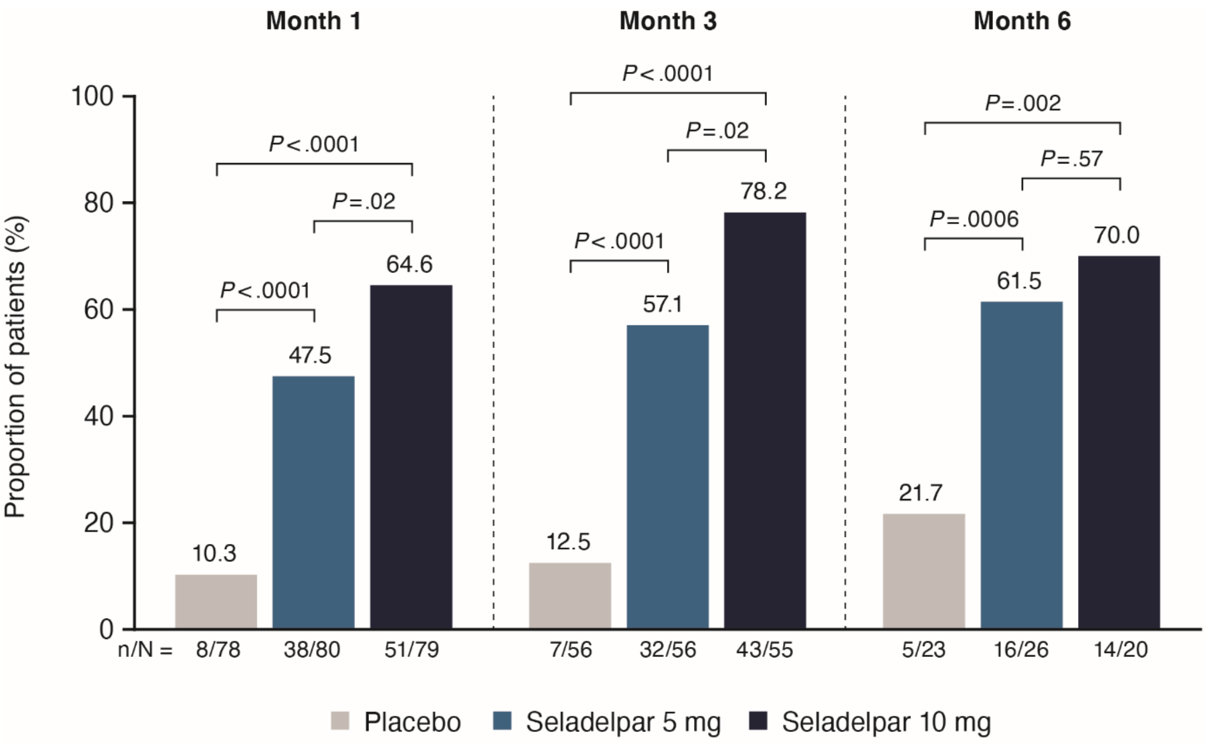

Composite endpoint was defined as ALP serum levels <1.67×ULN, ≥15% decrease in ALP serum levels, and total bilirubin serum levels ≤ULN.

P-values are based on the Cochran-Mantel-Haenszel test adjusted for both randomization stratification variables.

Note: Patients who discontinued treatment prior to time point due to reasons other than study termination and who did not have evaluable data at time point were considered nonresponders.

Abbreviations: ALP, alkaline phosphatase; N, number of patients in the treatment group; n, number of patients in the category; ULN, upper limit of normal.

Supplemental Figure S2. NRS change from baseline through Month 6

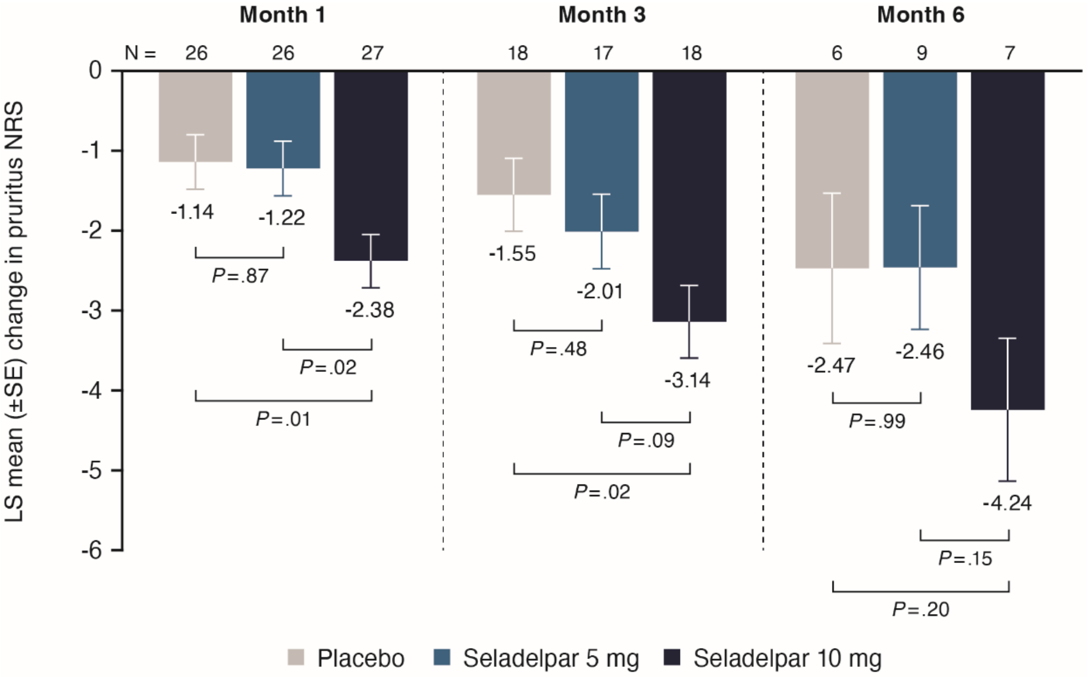

Population included only patients with pruritus NRS ≥4 at baseline.

Change from baseline was estimated by an ANCOVA model with treatment group and randomization ALP stratification factors as fixed effects and baseline pruritus score as a covariate.

Abbreviations: ALP, alkaline phosphatase; ANCOVA, analysis of covariance; LS, least squares; N, number of patients in the treatment group; NRS, numerical rating scale; SE, standard error.

Supplemental Figure S3. 5-D itch scale. Mean change from baseline and absolute scores through Month 3 for the 5-D itch scale degree domain (A-B), duration domain (C-D), direction domain (E-F), sleep domain (G-H), highest disability domain (I-J), and total score (K-L) among patients with baseline pruritus NRS ≥4

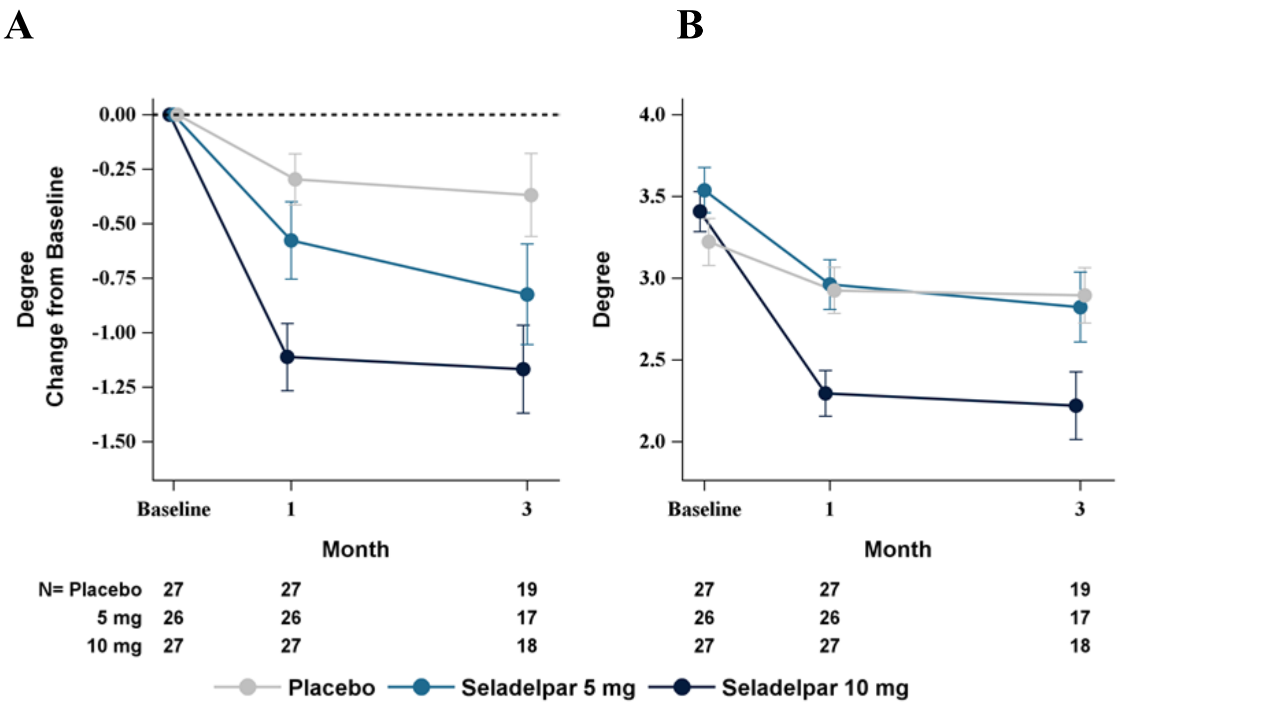

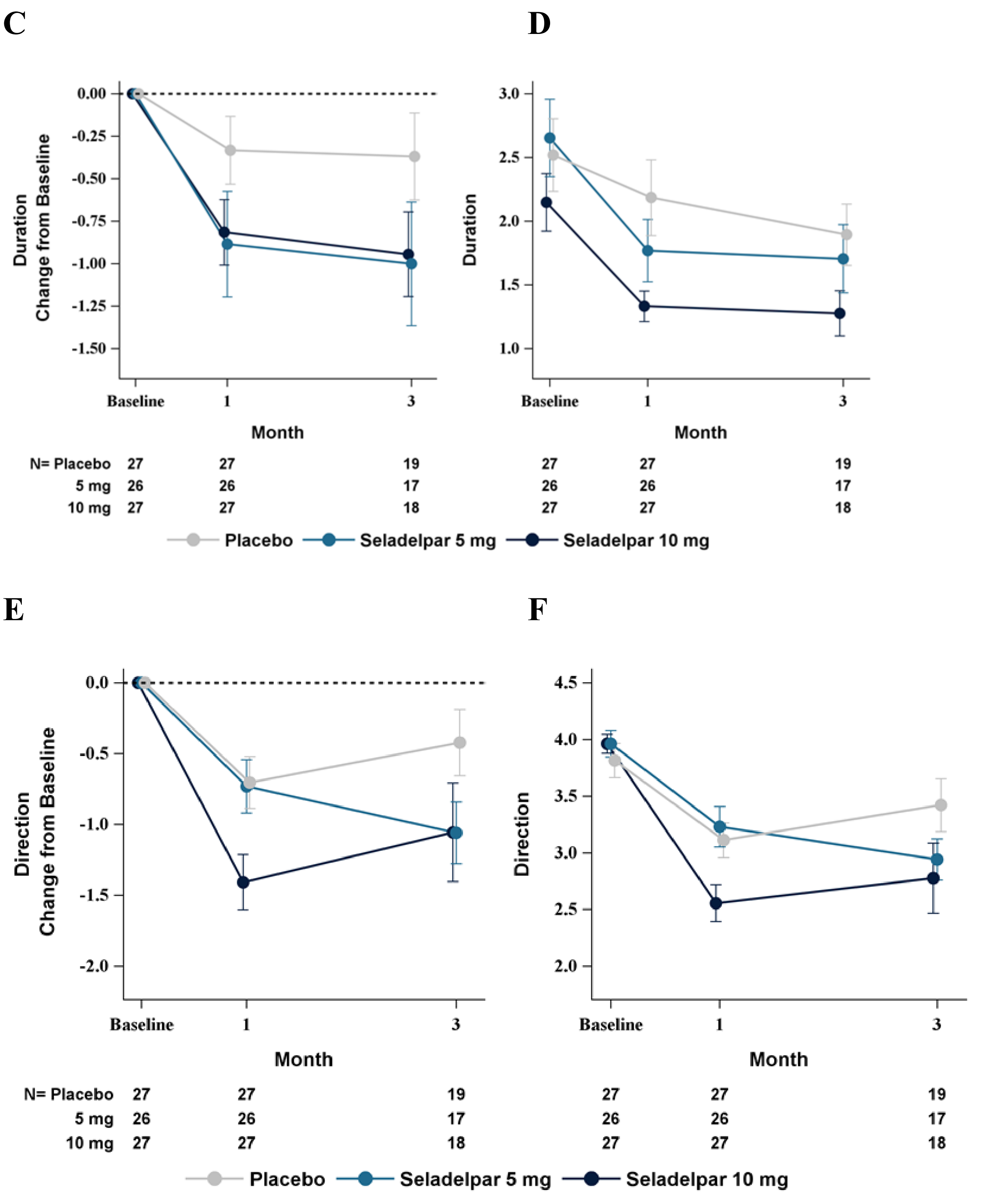
C
D
Duration Change from Baseline
0.00
-0.25
-0.50
-0.75
-1.00
-1.25
-1.50
Duration
3.0
2.5
2.0
1.5
1.0
Baseline
1
3
Month
N= Placebo 27 27 19
5 mg 26 26 17
10 mg 27 27 18
Placebo
Seladelpar 5 mg
Seladelpar 10 mg
E
F
Direction Change from Baseline
0.0
-0.5
-1.0
-1.5
-2.0
Direction
4.5
4.0
3.5
3.0
2.5
2.0
Baseline
1
3
Month
N= Placebo 27 27 19
5 mg 26 26 17
10 mg 27 27 18
Placebo
Seladelpar 5 mg
Seladelpar 10 mg

G H

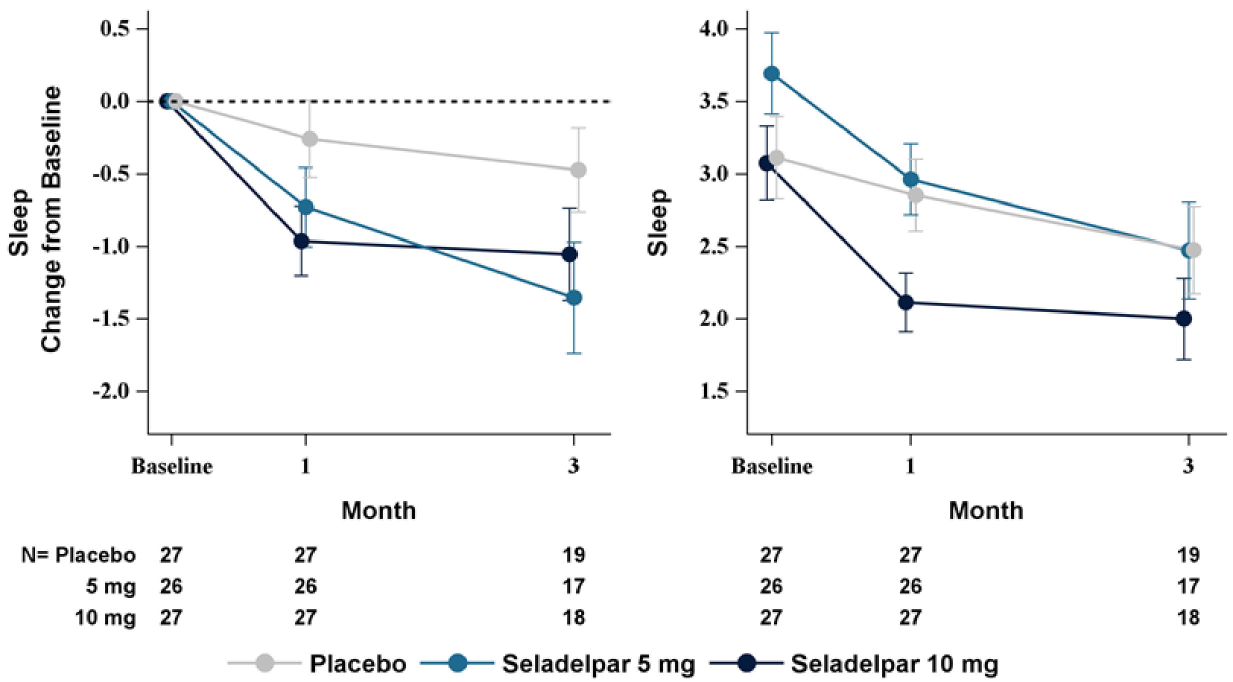
Sleep
Change from Baseline
0.5
0.0
-0.5
-1.0
-1.5
-2.0
Baseline
1
3
Month
Sleep
4.0
3.5
3.0
2.5
2.0
1.5
N= Placebo 27 27 19 27 27 19
5 mg 26 26 17 26 26 17
10 mg 27 27 18 27 27 18
Placebo
Seladelpar 5 mg
Seladelpar 10 mg

I J

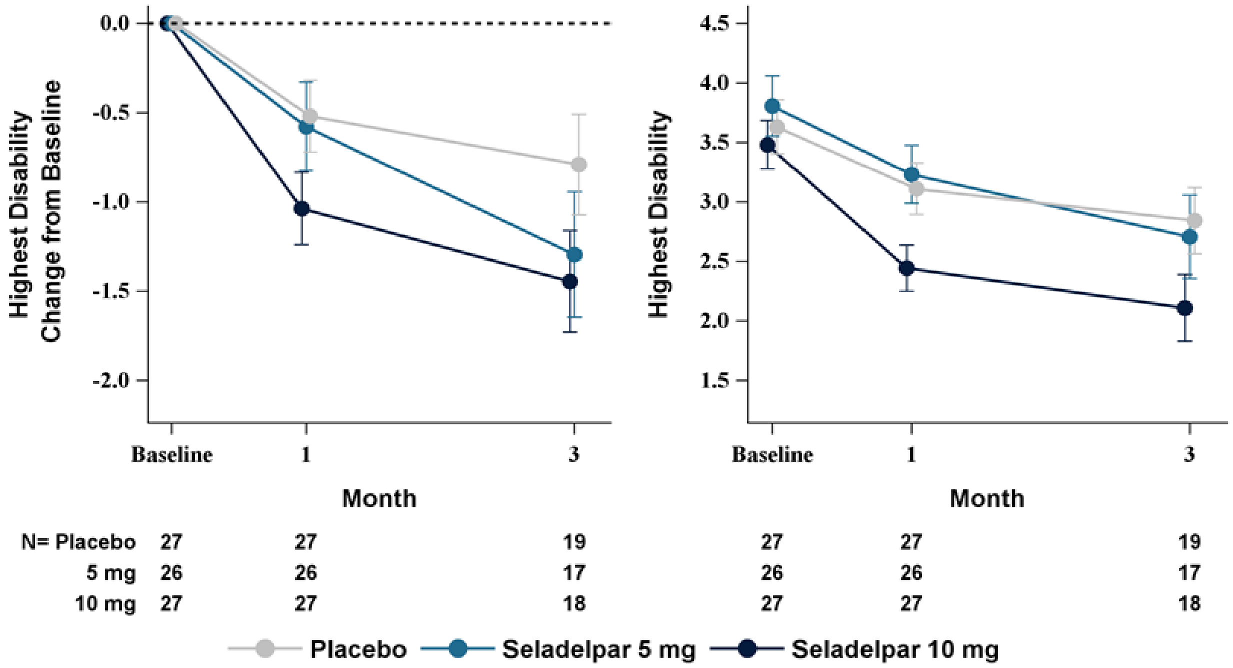
Highest Disability
Change from Baseline
0.0
-0.5
-1.0
-1.5
-2.0
Baseline
1
3
Month
Highest Disability
4.5
4.0
3.5
3.0
2.5
2.0
1.5
N= Placebo 27 27 19 27 27 19
5 mg 26 26 17 26 26 17
10 mg 27 27 18 27 27 18
Placebo
Seladelpar 5 mg
Seladelpar 10 mg

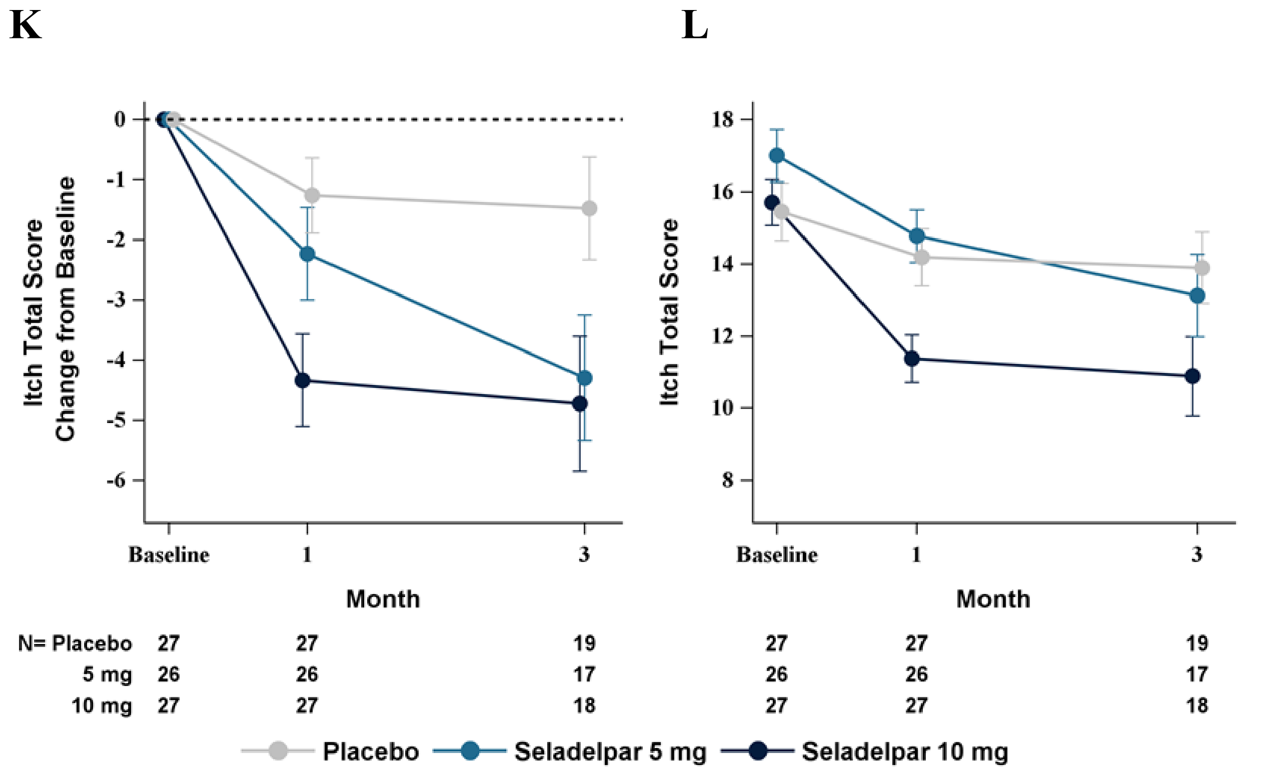

Abbreviations: BL, baseline; M, month; NRS, numerical rating scale; SE, standard error.

Supplemental Figure S4. PBC-40 questionnaire. Mean change from baseline and absolute scores through Month 3 in the PBC-40 questionnaire itch domain (A-B), cognitive domain (C-D), emotional domain (E-F), fatigue domain (G-H), symptoms domain (I-J), social domain (K-L), and total score (M-N) among patients with baseline pruritus NRS ≥4

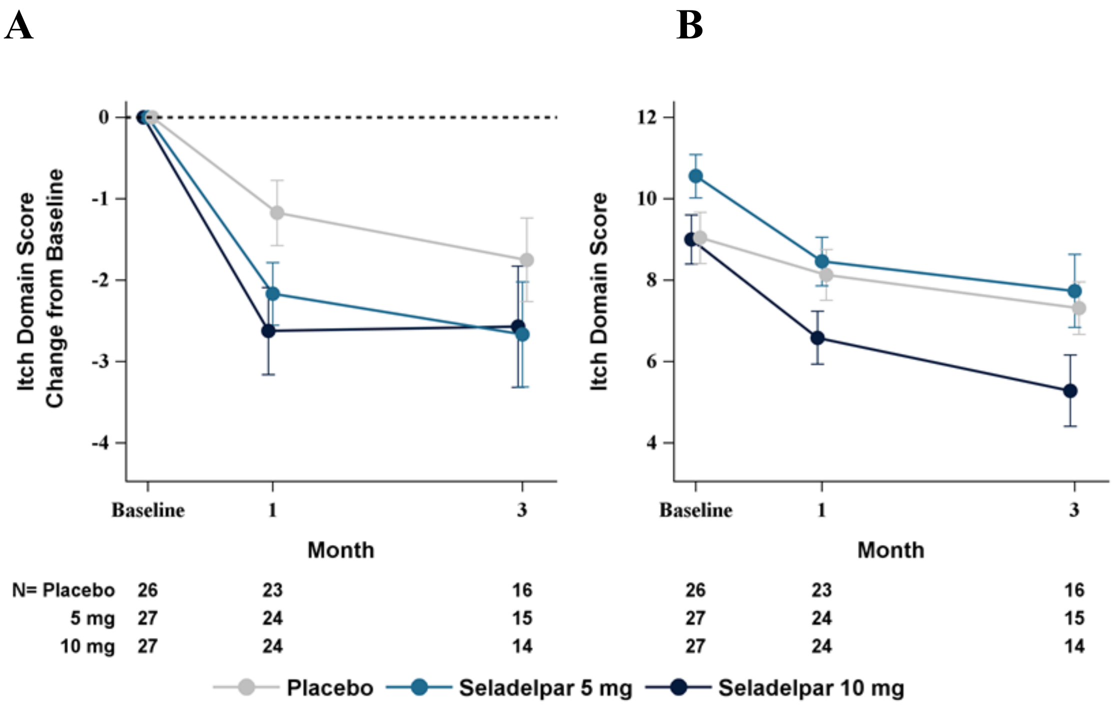

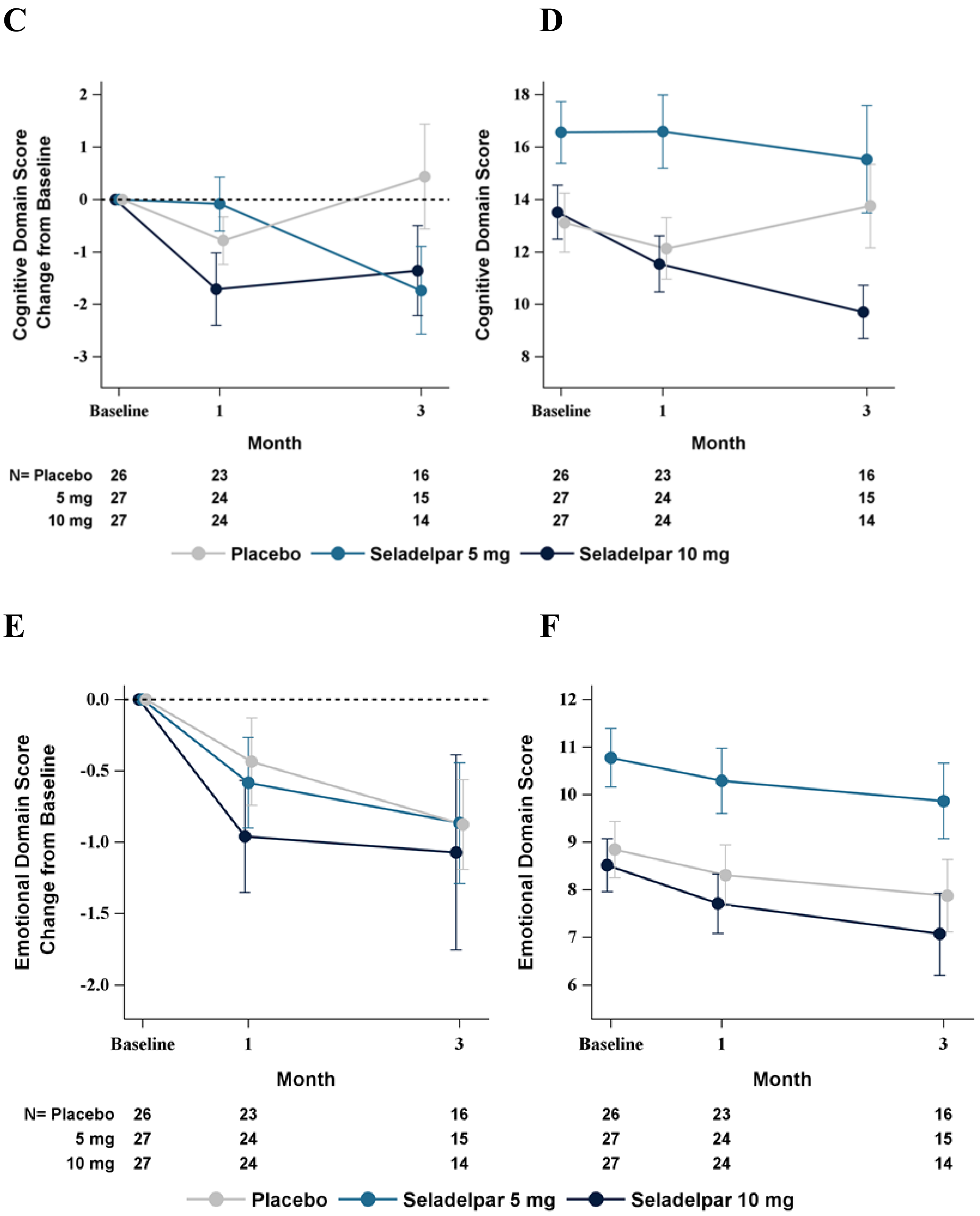
C
D
Cognitive Domain Score Change from Baseline
Cognitive Domain Score
Baseline
1
3
Month
N= Placebo 26 23 16
5 mg 27 24 15
10 mg 27 24 14
Placebo
Seladelpar 5 mg
Seladelpar 10 mg
E
F
Emotional Domain Score Change from Baseline
Emotional Domain Score
Baseline
1
3
Month
N= Placebo 26 23 16
5 mg 27 24 15
10 mg 27 24 14
Placebo
Seladelpar 5 mg
Seladelpar 10 mg

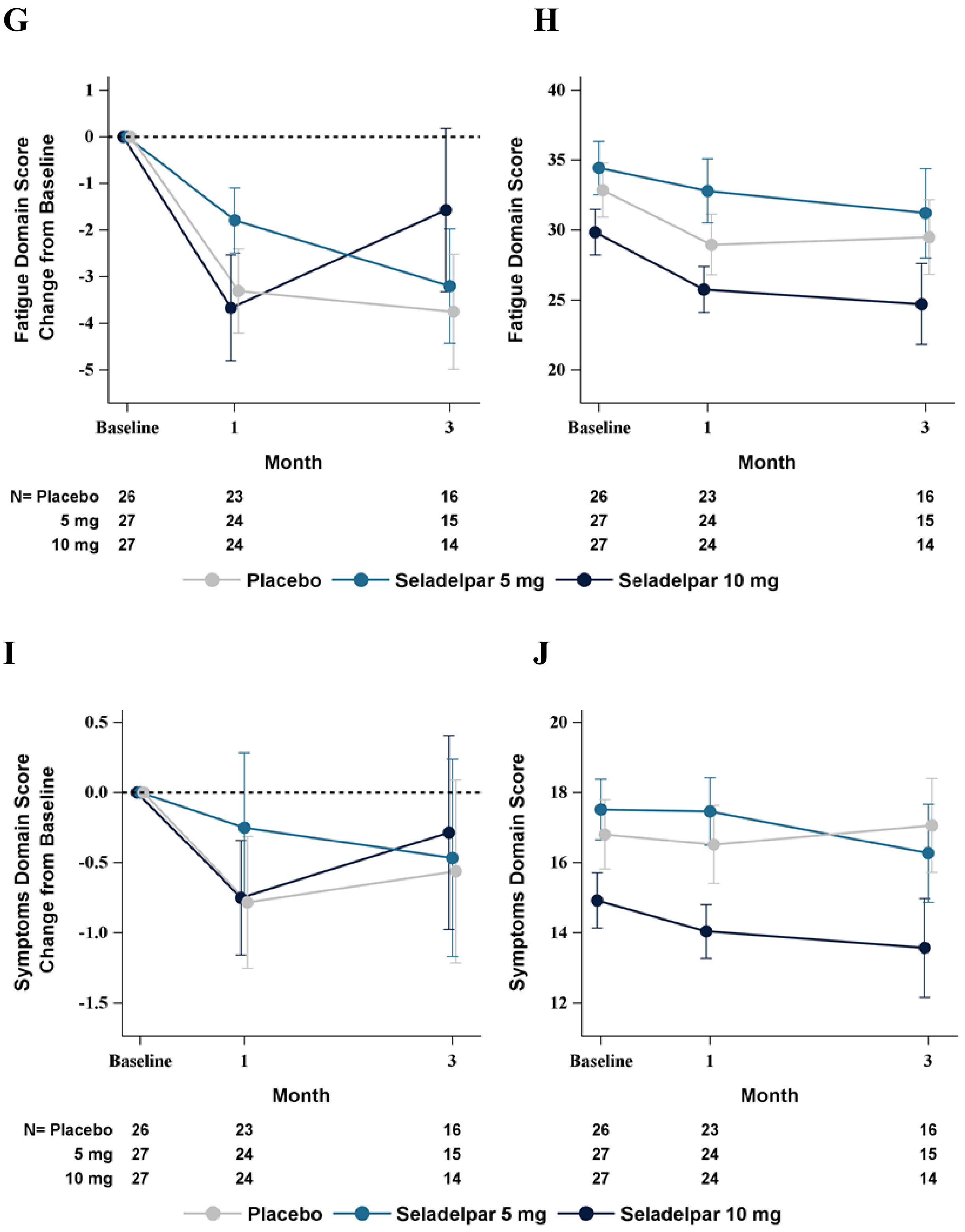
G
H
Fatigue Domain Score
Change from Baseline
1
0
-1
-2
-3
-4
-5
Fatigue Domain Score
40
35
30
25
20
Baseline
1
3
Month
N= Placebo 26 23 16 26 23 16
5 mg 27 24 15 27 24 15
10 mg 27 24 14 27 24 14
Placebo
Seladelpar 5 mg
Seladelpar 10 mg
I
J
Symptoms Domain Score
Change from Baseline
0.5
0.0
-0.5
-1.0
-1.5
Symptoms Domain Score
20
18
16
14
12
Baseline
1
3
Month
N= Placebo 26 23 16 26 23 16
5 mg 27 24 15 27 24 15
10 mg 27 24 14 27 24 14
Placebo
Seladelpar 5 mg
Seladelpar 10 mg

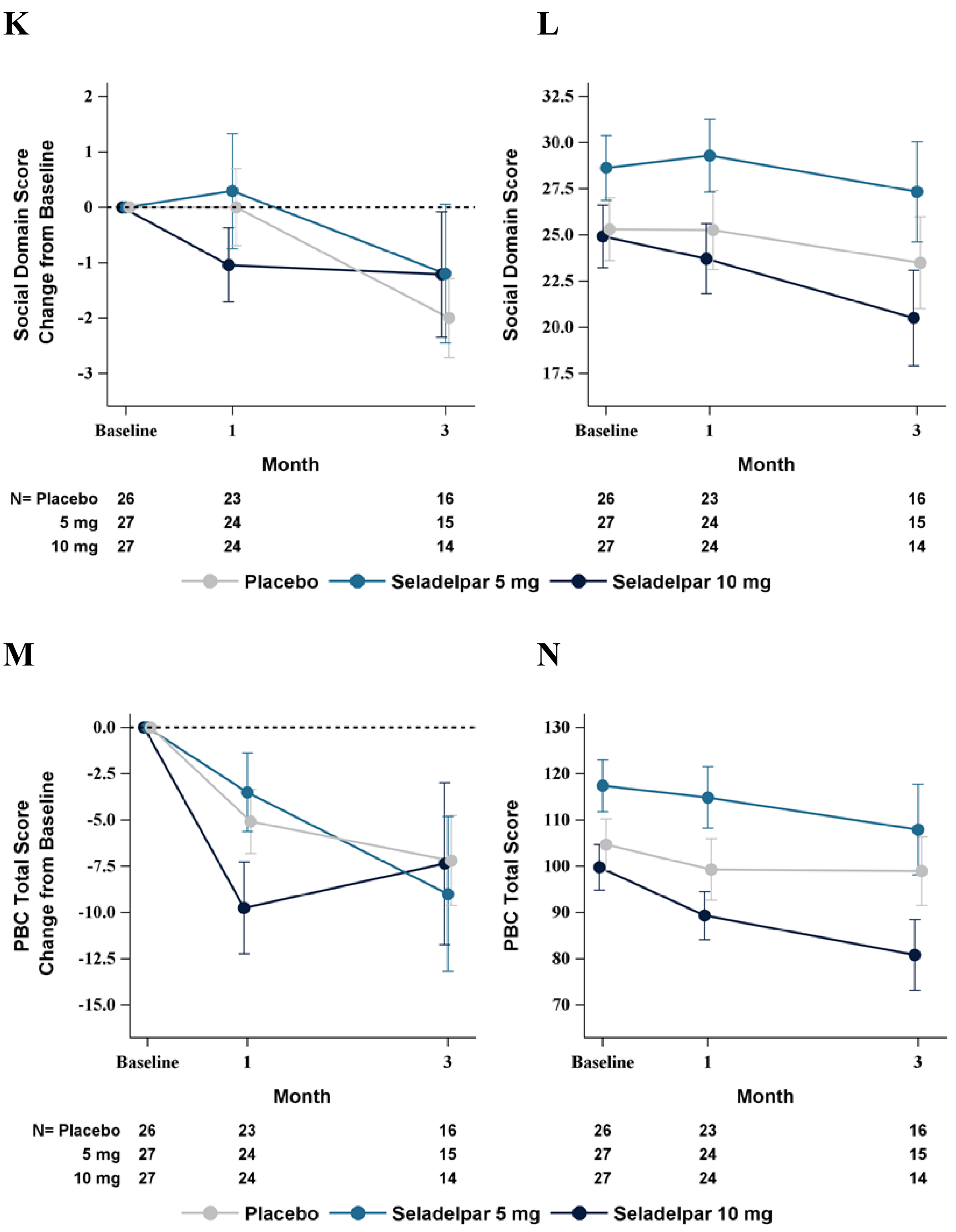

Abbreviations: BL, baseline; M, month; NRS, numeric rating scale; PBC, primary biliary cholangitis; SE, standard error.

Supplemental Figure S5. Relative changes from baseline and absolute serum levels of total (A-B), direct (C-D), and indirect bilirubin (E-F); AST (G-H); GGT (I-J); triglycerides (K-L); total cholesterol (M-N); LDL-C (O-P); and HDL-C (Q-R) through Month 6

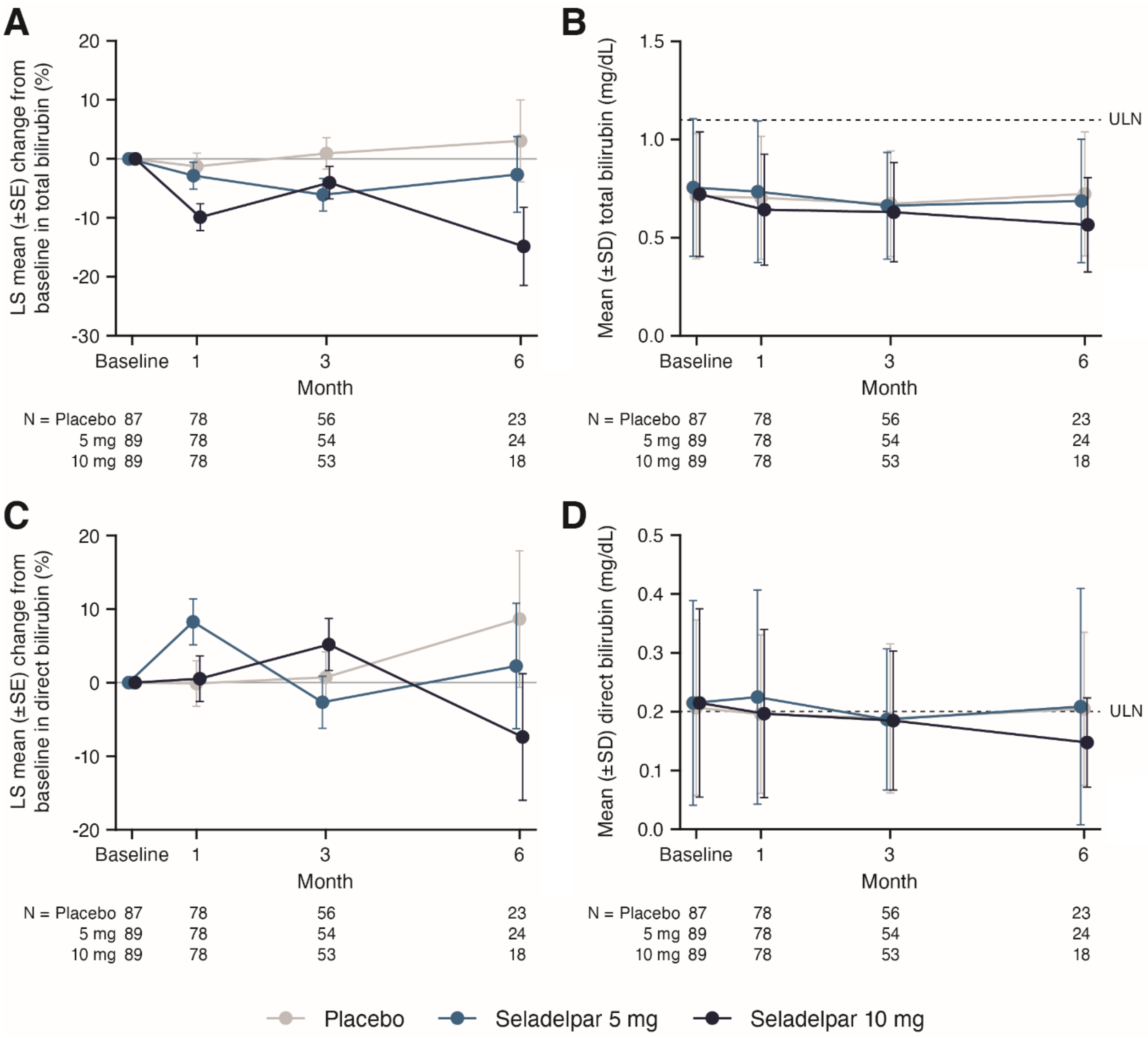

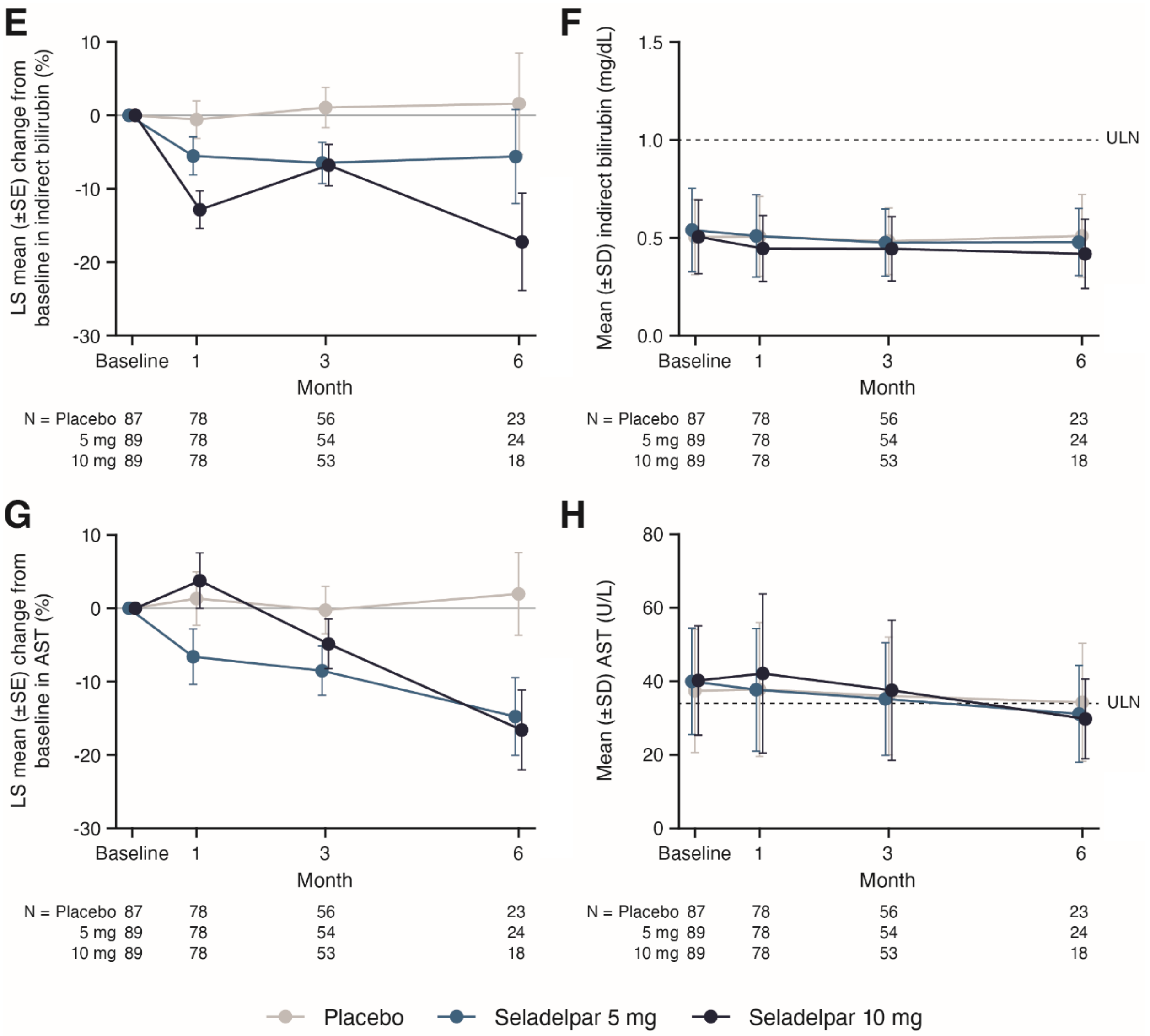

E
LS mean (±SE) change from baseline in indirect bilirubin (%)
10
0
-10
-20
-30
Baseline
1
3
6
Month
N = Placebo 87 78 56 23
5 mg 89 78 54 24
10 mg 89 78 53 18
F
Mean (±SD) indirect bilirubin (mg/dL)
1.5
1.0
0.5
0.0
ULN
Baseline
1
3
6
Month
N = Placebo 87 78 56 23
5 mg 89 78 54 24
10 mg 89 78 53 18
G
LS mean (±SE) change from baseline in AST (%)
10
0
-10
-20
-30
Baseline
1
3
6
Month
N = Placebo 87 78 56 23
5 mg 89 78 54 24
10 mg 89 78 53 18
H
Mean (±SD) AST (U/L)
80
60
40
20
0
ULN
Baseline
1
3
6
Month
N = Placebo 87 78 56 23
5 mg 89 78 54 24
10 mg 89 78 53 18
Placebo
Seladelpar 5 mg
Seladelpar 10 mg

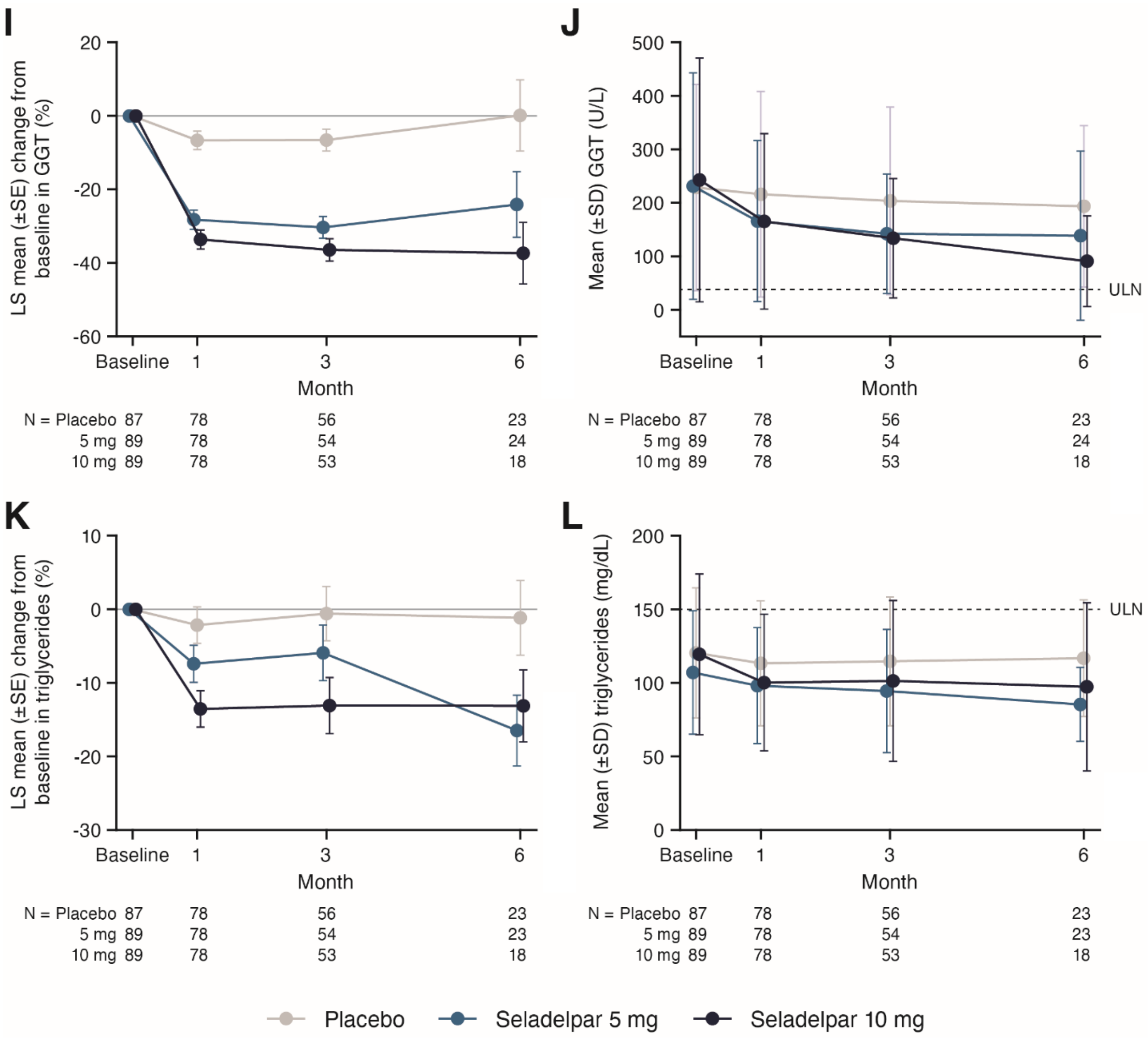
I
LS mean (±SE) change from baseline in GGT (%)
20
0
-20
-40
-60
Baseline 1 3 6
Month
N = Placebo 87 78 56 23
5 mg 89 78 54 24
10 mg 89 78 53 18
J
Mean (±SD) GGT (U/L)
500
400
300
200
100
0
ULN
Baseline 1 3 6
Month
N = Placebo 87 78 56 23
5 mg 89 78 54 24
10 mg 89 78 53 18
K
LS mean (±SE) change from baseline in triglycerides (%)
10
0
-10
-20
-30
Baseline 1 3 6
Month
N = Placebo 87 78 56 23
5 mg 89 78 54 23
10 mg 89 78 53 18
L
Mean (±SD) triglycerides (mg/dL)
200
150
100
50
0
ULN
Baseline 1 3 6
Month
N = Placebo 87 78 56 23
5 mg 89 78 54 23
10 mg 89 78 53 18
Placebo
Seladelpar 5 mg
Seladelpar 10 mg

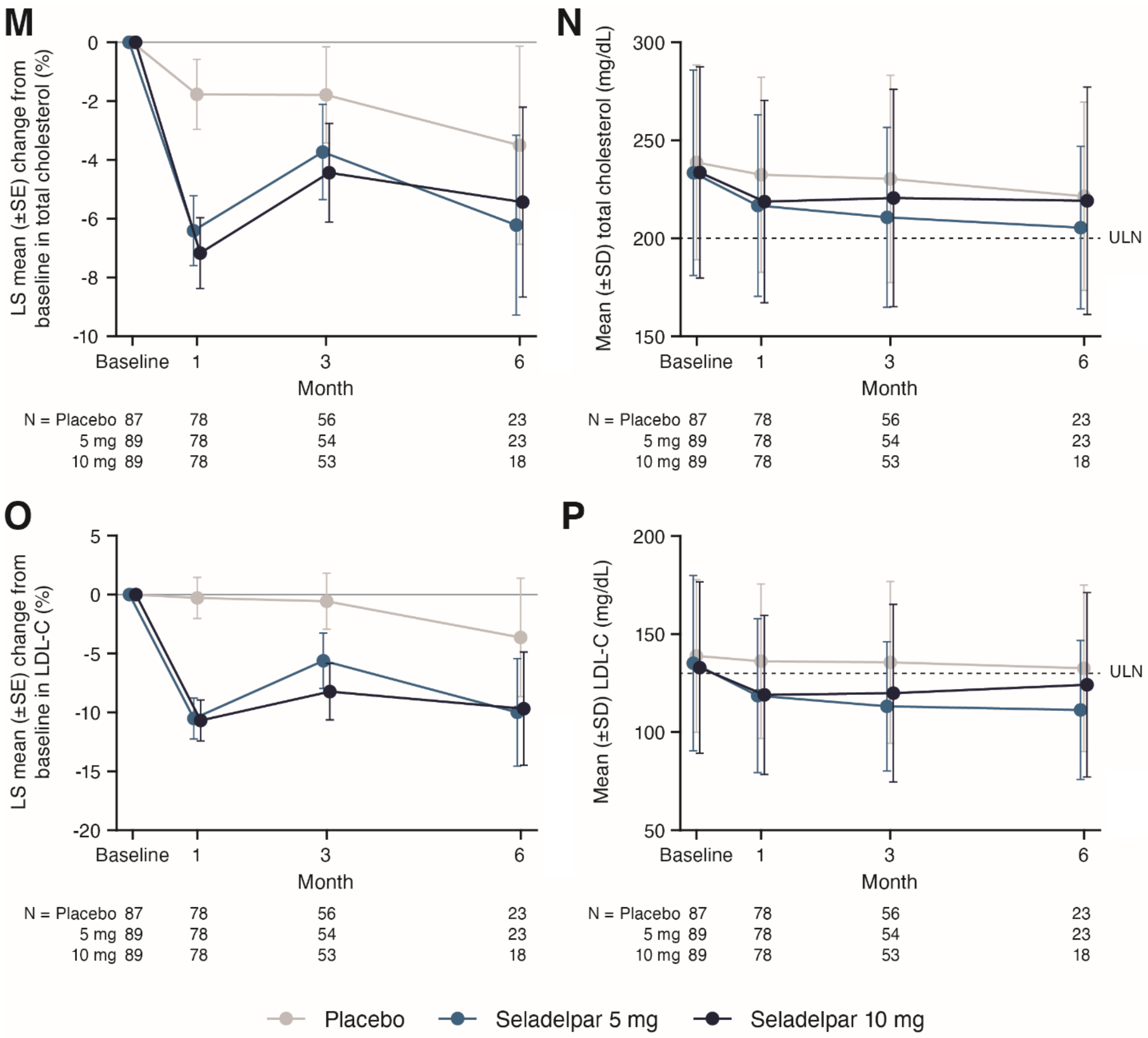
M
LS mean (±SE) change from baseline in total cholesterol (%)
N
Mean (±SD) total cholesterol (mg/dL)
ULN
O
LS mean (±SE) change from baseline in LDL-C (%)
P
Mean (±SD) LDL-C (mg/dL)
ULN
Baseline 1 3 6
Month
N = Placebo 87 78 56 23
5 mg 89 78 54 23
10 mg 89 78 53 18
Placebo
Seladelpar 5 mg
Seladelpar 10 mg

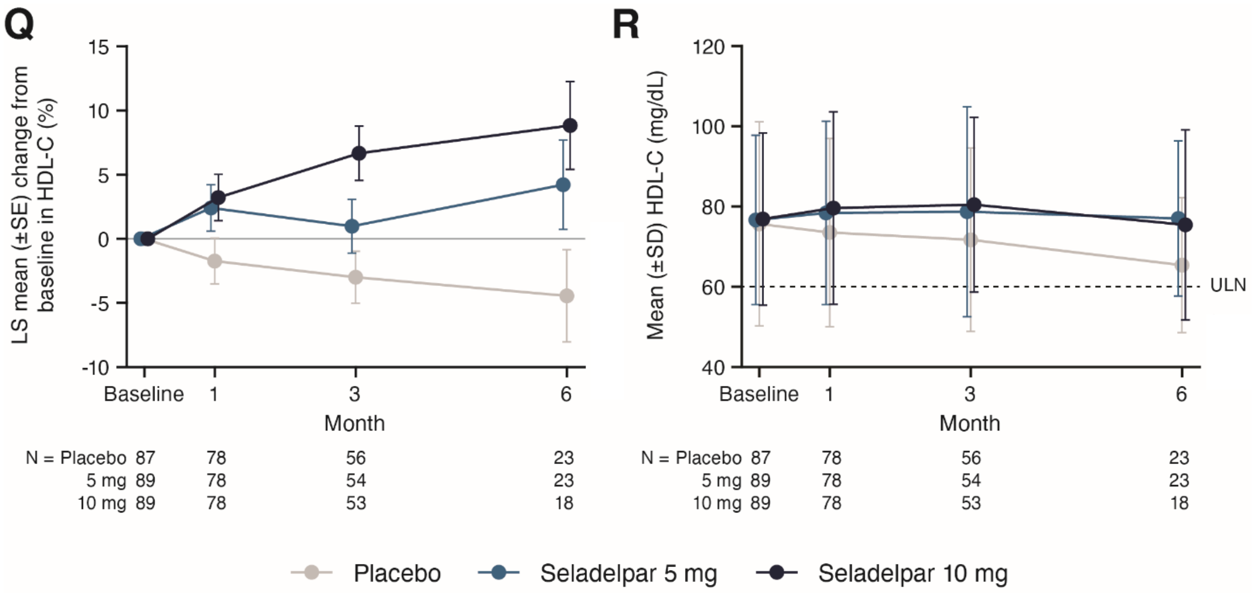

Percent change from baseline was estimated by ANCOVA with percent change from baseline as the dependent variable, treatment group and randomization stratification factors as fixed effects, and baseline as a covariate.

Abbreviations: ANCOVA, analysis of covariance; AST, aspartate aminotransferase; GGT, gamma glutamyl transferase; HDL-C, high-density lipoprotein cholesterol; LDL-C, low-density lipoprotein cholesterol; N, number of patients in the treatment group; SD, standard deviation; SE, standard error.
